# Plant Breeding for Intercropping in Temperate Field Crop Systems: A Review

**DOI:** 10.3389/fpls.2022.843065

**Published:** 2022-03-31

**Authors:** Virginia M. Moore, Brandon Schlautman, Shui-zhang Fei, Lucas M. Roberts, Marnin Wolfe, Matthew R. Ryan, Samantha Wells, Aaron J. Lorenz

**Affiliations:** ^1^Plant Breeding and Genetics Section, School of Integrative Plant Science, Cornell University, Ithaca, NY, United States; ^2^The Land Institute, Salina, KS, United States; ^3^Department of Horticulture, Iowa State University, Ames, IA, United States; ^4^Department of Agronomy and Plant Genetics, University of Minnesota, Saint Paul, MN, United States; ^5^Department of Crop, Soil and Environmental Sciences, College of Agriculture, Auburn University, Auburn, AL, United States; ^6^Soil and Crop Sciences Section, School of Integrative Plant Science, Cornell University, Ithaca, NY, United States

**Keywords:** agroecology, ecosystem services, intercropping, plant breeding, polyculture, sustainable cropping systems

## Abstract

Monoculture cropping systems currently dominate temperate agroecosystems. However, intercropping can provide valuable benefits, including greater yield stability, increased total productivity, and resilience in the face of pest and disease outbreaks. Plant breeding efforts in temperate field crops are largely focused on monoculture production, but as intercropping becomes more widespread, there is a need for cultivars adapted to these cropping systems. Cultivar development for intercropping systems requires a systems approach, from the decision to breed for intercropping systems through the final stages of variety testing and release. Design of a breeding scheme should include information about species variation for performance in intercropping, presence of genotype × management interaction, observation of key traits conferring success in intercropping systems, and the specificity of intercropping performance. Together this information can help to identify an optimal selection scheme. Agronomic and ecological knowledge are critical in the design of selection schemes in cropping systems with greater complexity, and interaction with other researchers and key stakeholders inform breeding decisions throughout the process. This review explores the above considerations through three case studies: (1) forage mixtures, (2) perennial groundcover systems (PGC), and (3) soybean-pennycress intercropping. We provide an overview of each cropping system, identify relevant considerations for plant breeding efforts, describe previous breeding focused on the cropping system, examine the extent to which proposed theoretical approaches have been implemented in breeding programs, and identify areas for future development.

## Introduction

Crop diversity provides an array of benefits (Altieri, 1999; Letourneau et al., 2011) and can appear at multiple spatial and temporal scales (e.g., across landscapes, seasons, farms, or fields). Intercropping represents within-field diversity, is defined as growing two or more crop species simultaneously in the same field, and encompasses a range of practices including mixed intercropping (growing component crops simultaneously with no distinct row arrangement), row intercropping (growing component crops simultaneously in different rows), strip intercropping (growing component crops simultaneously in different strips), and relay intercropping (growing component crops with overlapping growth periods; Andrews and Kassam, 1976). Intercropping can provide valuable benefits, including increased yield (Trenbath, 1974; Hauggaard-Nielsen et al., 2001; Nyfeler et al., 2009; Finn et al., 2013; Martin-Guay et al., 2018; Li et al., 2020), yield stability (Rao and Willey, 1980; Raseduzzaman and Jensen, 2017), improved crop quality (Sleugh et al., 2000; Bélanger et al., 2014), reduced pest and disease impacts (Altieri, 1999; Boudreau, 2013; Gaba et al., 2015), improved weed management (Hauggaard-Nielsen et al., 2001; Finn et al., 2013; Johnson et al., 2017; Connolly et al., 2018; Hoerning et al., 2020), reduced input needs (Nyfeler et al., 2009; Gaba et al., 2015; Raskin et al., 2017), improved soil health (Cong et al., 2015; Li et al., 2021), support for a wide range of native pollinators (Eberle et al., 2015; Forcella et al., 2021), and a range of other ecosystem services, such as wildlife conservation, soil conservation, water quality improvements (Weyers et al., 2021), and carbon sequestration (Malézieux et al., 2009). Intraspecific diversity in the form of cultivar mixtures can provide benefits for productivity and resilience (Reiss and Drinkwater, 2018), but this review focuses on interspecific diversity through intercropping.

Intercropping is an ancient practice that has been used for thousands of years and remains an important practice in many parts of Africa, Asia, and Latin America. However, temperate regions have seen shifts away from intercropping and toward monoculture production, which is associated with greater mechanization, specialization, and input use (Anil et al., 1998; Altieri, 1999; Crews and Peoples, 2004). Intercropping systems are generally seen as labor-intensive and incompatible with mechanization and the need for standardized products (Brooker et al., 2015). However, with the array of environmental problems associated with modern agriculture (Foley et al., 2005; Pretty et al., 2018), there is interest among researchers and farmers in increasing diversity in cropping systems (Arbuckle and Roesch-McNally, 2015; Mortensen and Smith, 2020), and with new technological advances (e.g., new machinery, precision agriculture technology, and genomic tools), there are new possibilities of developing intercropping systems for modern agriculture in temperate field crops (Brooker et al., 2015). Modern plant breeding efforts in temperate field crops have primarily focused on monoculture production (Henkhaus et al., 2020). Still, as interest in intercropping for temperate agriculture increases, cultivars must be adapted to these cropping systems.

Experimental approaches and breeding schemes to improve germplasm for intercropping have been widely studied (Keller, 1946; Hamblin et al., 1976; Mead and Willey, 1980; Wright, 1985; Hill, 1990; Brooker et al., 2015), yet critical knowledge gaps exist that prevent greater utilization of intercropping. We briefly review the relevant literature in ecology, agronomy, and plant breeding and describe core experimental, breeding, and cropping system design approaches, and apply these core concepts to temperate field crops in three case studies of intercropping systems at various stages of breeding research and development: (1) forage mixtures, (2) perennial groundcover (PGC) systems, and (3) intercropping with winter oilseeds. Case studies were selected to represent a range of temporal and spatial interactions, agronomic and ecosystem service goals, and maturity of breeding efforts. Through these case studies, we examine the extent to which proposed theoretical approaches have been implemented in breeding programs and identify areas for future development.

### Defining the Problem and Solution Spaces

**Figure 1:**
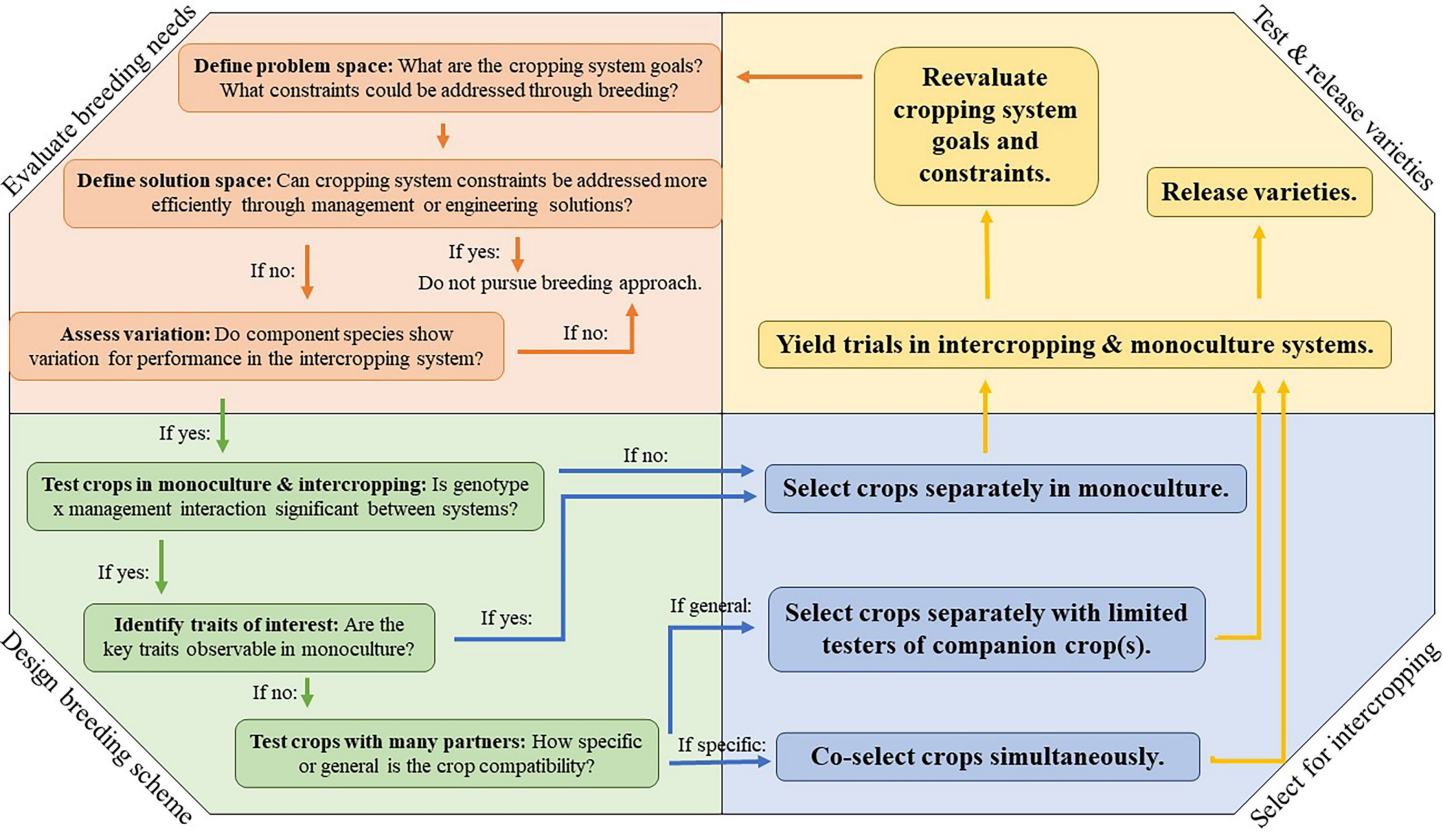
The process of breeding for intercropping systems.

When a plant breeder considers whether to breed for intercropping systems, they first need to identify the cropping system goals, and whether major cropping system constraints can be addressed through plant breeding (Figure 1). The possible benefits of planting crops in an intercropping system are diverse and support agronomic goals and ecosystem services (Tamburini et al., 2020). The relative importance of agronomic and ecosystem service goals varies by cropping system, and likewise, the relative contribution of each crop component toward those goals will vary. Some intercrops are planted to maximize short-term profitability by increasing productivity or quality, while others are used for their regulating and supporting ecosystem services (e.g., cover crop mixtures).

In systems where all crop components are harvested as cash crops, and in which the components are of relatively similar value, the main goal is often to increase total productivity of the system. However, if regulating and supporting ecosystem services are goals of the intercropping system, the main goal may be to balance tradeoffs between short-term profitability and benefits that might only manifest over longer periods or under certain circumstances. In this scenario, the system goals will likely be focused on the productivity of the primary crop, as ecosystem services or yield provided by the secondary crop must be achieved without compromising the primary crop. Programs can focus on avoiding yield or quality reductions in the primary crop by breeding for differential resource-use relative to the secondary crop. Alternatively, breeding programs may focus on adapting a secondary crop to use resources not needed for the primary crop, better tolerate the stress imposed by the primary, or even to facilitate the primary crop. Depending on the specific goal of the system, selection may take place within one or more component species, and selection criteria may be based on the total productivity of combined crop components or based on maximizing the productivity of a single component species.

When developing intercropping systems for temperate field crops, breeding decisions, and overall cropping system design will depend on these goals and the role each component crop plays in their realization. Both plant breeders and agronomists work to design and improve intercropping systems, and as plant breeders identify potential breeding goals, particularly in novel and complex intercropping systems, it is critical they engage with both agronomists and end users to identify breeding needs. Because plant breeding is a resource-intensive endeavor, it is also prudent for plant breeders to explore alternative approaches to improving intercropping system performance. This could include cropping system management (e.g., altering plant spacing, timing, or fertility) or engineering (e.g., adapting planting or harvesting equipment for multiple species) solutions. Agronomists, engineers, and other specialists should be engaged in this process of “defining the solution space” (Figure 1).

### Assessing Variation and Genotype × Management Interaction

Identifying meaningful variation within the target species is a prerequisite for crop improvement efforts. Early studies often include screening diverse germplasm for performance in intercropping (Wright, 1985; Haug et al., 2021). In addition, to determine whether an intercropping-specific breeding program is merited, it is important to determine whether genotypes show differential performance in monoculture compared to intercropping systems. If genotype × management (GxM) interactions are not significant, then genotypes selected in monoculture can be used in intercropping systems (Annicchiarico et al., 2019). However, in the presence of significant rank changes, there is a need for intercropping-specific breeding efforts. To evaluate the significance of GxM interactions, diverse germplasm should be evaluated in both monoculture and intercropping systems (Brooker et al., 2015). Such comparative studies allow for the calculation of variance components, correlations, and heritabilities (Annicchiarico, 2003), and can inform breeder decisions about breeding methods and whether mixture-focused breeding efforts are required.

### Performance in Intercropping: Competition and Overyielding

When designing a selection scheme for intercropping systems, a major question is which traits should be considered in the selection process? Ecological theory can provide insights to understand interspecies interactions and productivity in these systems (Li et al., 2014; Brooker et al., 2015; Litrico and Violle, 2015). While intercropping systems provide numerous environmental and agronomic benefits, competition between component species can reduce productivity. Competition between component species may come in the form of exploitation competition (competition for the same resources such as light, water, or nutrients) or interference competition (directly altering the resource acquisition behavior of another organism; Case and Gilpin, 1974; Schoener, 1983).

Intercropping systems are often challenged by asymmetric exploitation competition, such that one species has a competitive advantage over another, which can reduce productivity and overall benefits of the system (Connell, 1983; Thomas, 1992; Corre-Hellou et al., 2006; Bybee-Finley et al., 2016). The competitive advantage may depend on growing conditions; for example, low N availability favors legumes over other plants, and moisture limitations in arid regions may favor one component species over another. Such genotype × environment (GxE) and genotype × environment × management (GxExM) interactions will inform the regional focus and breeding approaches within intercropping breeding programs. Competition between partners may also change over time, depending on the phenology, stress tolerance, and persistence of component crops (Raskin et al., 2017; Ginakes et al., 2020). Temporal dynamics play a role in both cropping system design and breeding. For example, understanding key growth periods during which competition will be more detrimental (e.g., through modeling yield loss due to competition) may help to select appropriate crop pairings and determine breeding objectives (e.g., early maturity; Gaudio et al., 2019; Cheriere et al., 2020; Bourke et al., 2021; Schlautman et al., 2021).

Allelopathy, or chemical inhibition of one plant by another, is a common form of interference competition. Allelopathic ability has been a focus for breeding programs with an interest in weed suppression, and screening and selection for allelopathy have been conducted in cereals and other crop species, including rice, wheat, barley, oat, rye, cassava, sunflower, and sorghum (Worthington and Reberg-Horton, 2013). In the context of intercropping systems, the role of allelopathy may be important depending on the component species, and selection criteria may include reduced allelochemical production or reduced susceptibility to the allelochemicals produced by the partner species. In general, asymmetric competition may be more or less important depending on the goals of the system. For example, when one crop is planted primarily for ecosystem services, farmers may be less willing to compromise yield of its cash crop partner, whereas in intercropping systems involving two cash crops of comparable value, some yield reduction of each component may be acceptable.

**Figure 2:**
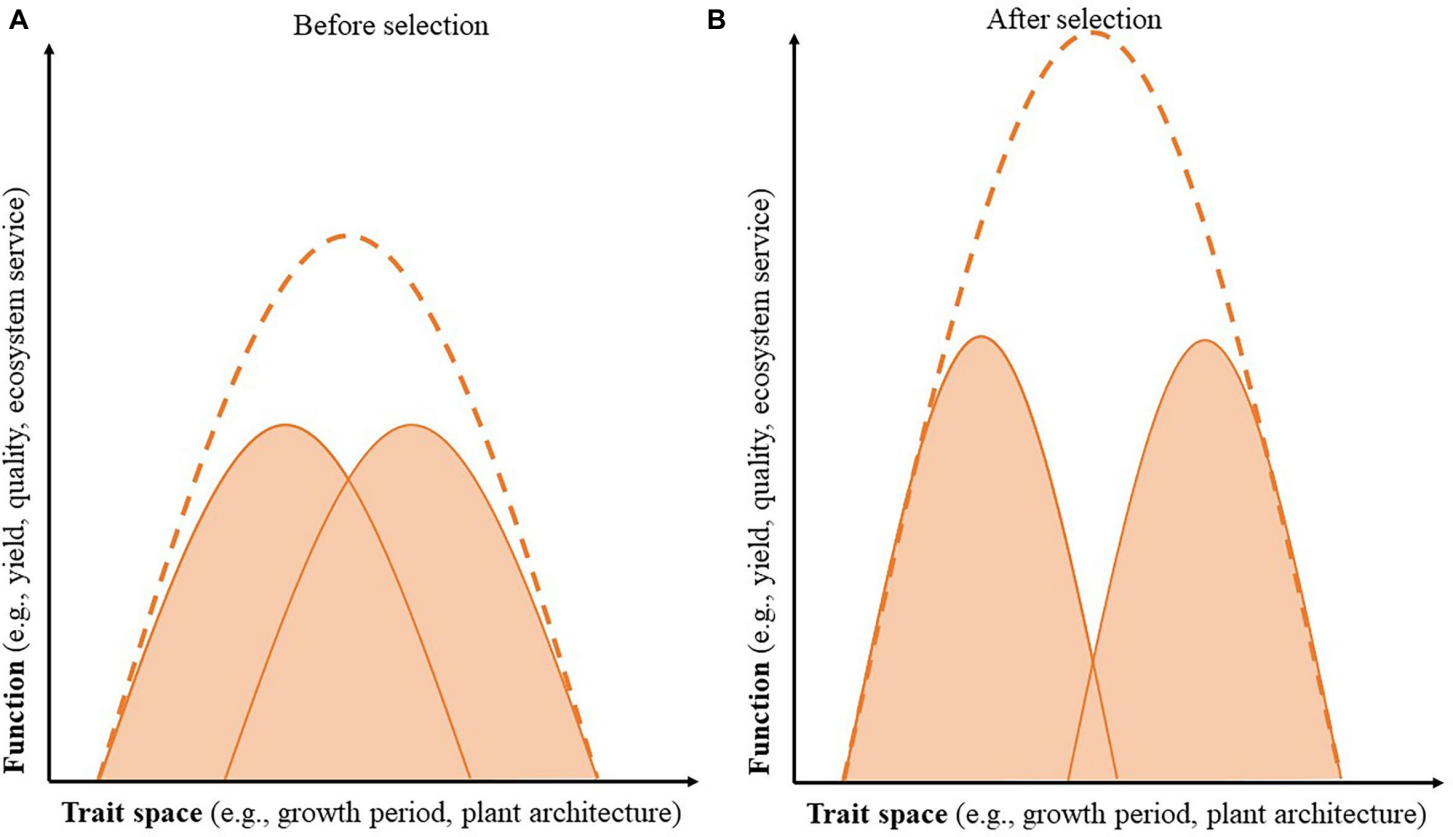
Selecting component species for niche differentiation enhances the combined function of the intercropping system. The x-axis represents the trait space, which may represent a temporal (e.g., growth period and maturity date), spatial (e.g., rooting depth and plant height), or other niche. The y-axis represents the desired cropping system function, including crop yield or quality, or a range of ecosystem services. The shaded areas represent each component species, and the dotted line represents the combined performance of the intercropping system. Before selection **(A)** there is more competition between component species (or overlap between the two curves), and after selection for niche differentiation **(B)** there is decreased competition between the component species, allowing for greater combined function of the cropping system.

Overyielding, or increased productivity in more diverse natural and agricultural ecosystems, often occurs in intercropping systems and can be explained through complementary interactions such as niche differentiation and facilitation. Niche differentiation is the process by which sympatric species avoid competitive interactions by differentially using resources; it can lead to greater productivity in natural and agricultural systems through increased resource acquisition and reduces interspecific competition (Hector et al., 1999; Fargione and Tilman, 2005; Hauggaard-Nielsen and Jensen, 2005; Li et al., 2007, 2014; Litrico and Violle, 2015). Previous research shows that, when designing and breeding for intercropping systems, an overarching goal should be to increase niche differentiation as a way to reduce competition between component crops and increase overall productivity of the system (Figure 2; Li et al., 2014; Brooker et al., 2015; Litrico and Violle, 2015; Annicchiarico et al., 2019). Niche differentiation may occur either spatially or temporally. For example, a focus on rooting depth or above-ground plant architecture could differentiate the resource space exploited by each component crop, whereas a focus on phenology could differentiate crops based on the period of maximum growth (Litrico and Violle, 2015). Increasing phenotypic plasticity could also contribute to species complementarity by increasing niche differentiation when planted in intercropping systems (Zhu et al., 2015).

According to Callaway (1995), facilitation, or positive interactions among plants, can occur directly, for example, by reducing environmental stress or increasing resource availability. Facilitation can also occur indirectly through elimination of competitors (e.g., through allelopathic effects on susceptible weeds), promoting other beneficial organisms, or providing protection from herbivores. Both niche differentiation and facilitation contribute to increased productivity in intercropping systems and can be difficult to distinguish in practice (Loreau and Hector, 2001). Facilitation has been observed in intercropping systems through mechanisms, such as improved nutrient availability (e.g., nitrogen fixation or mobilization of other nutrients), modification of root architecture, and suppression of pests and diseases (Hauggaard-Nielsen and Jensen, 2005; Li et al., 2014). Facilitation is more likely to occur with perennial intercrops compared to annual systems, since annuals have a more limited time to see beneficial effects from their partner species (Bybee-Finley and Ryan, 2018). Selecting for facilitation-related traits may therefore be more fruitful in perennials than annuals.

### Designing the Breeding Scheme

One challenge, in practice, is to identify traits that are straightforward to phenotype and have high correlation with intercropping performance. If these traits are known, highly correlated and observable without intercropping, then it will be possible to select component crops in monoculture. This is known as a trait-informed approach. Otherwise, it may be more efficient to select directly in an intercropping system, otherwise known as a trait-blind approach (Barot et al., 2017). Preliminary experiments are required, first to determine the appropriateness of the trait-informed or trait-blind approach by evaluating candidate traits in both mono- and mixed cropping (Brooker et al., 2015; Annicchiarico et al., 2019). If traits can be identified that are observable under monoculture, which have a sufficiently strong genetic correlation, it may be most efficient to select indirectly for intercropping performance based on monoculture data (Atlin and Frey, 1989; Brummer, 2006). Indirect selection can be more effective than direct selection when the heritability of the trait is larger in an off-target environment (monoculture system) than in an on-target environment (intercropped system; Holland and Brummer, 1999; Bänziger and Cooper, 2001; Brummer, 2006). Selecting in monoculture may also be desirable when intercropping involves more complicated management including narrower windows of operation or greater precision for weed and nutrient management.

When breeding for systems with multiple species, the number of combinatorial interactions can quickly become impractical experimentally. The concepts of general mixing ability (GMA) and specific mixing ability (SMA) introduced by Wright (1985) (also referred to by Hill, 1990 as general and specific ecological combining ability) have been established to understand the necessity of recurrent selection and variety development for each component intercrop. The GMA and SMA are analogous to the classical concepts of general and specific combining ability (Sprague and Tatum, 1942; Griffing, 1956) and have also been applied to understand variety mixtures and multilines (Dawson and Goldringer, 2011). Genotypes with high GMA would perform well in a wide range of intercropping scenarios regardless of the identity of their partner, whereas high SMA and low GMA indicate good performance with another specific genotype but lack of general adaptation to many intercropping systems. In the ideal scenario, GMA would have a larger contribution relative to SMA, allowing the breeding program to test material with a narrow set of entries (Annicchiarico et al., 2019). Temporal rotation and spatial intercropping systems might often be targets for focus on improving GMA, since breeders’ products are expected to be paired in the field with varieties chosen by growers. However, breeders developing mixtures in contexts where they may control the varietal combinations employed by farmers will potentially be able to exploit SMA.

Despite the importance of determining the relative importance of GMA and SMA to optimize efficiency of intercropping-focused breeding programs, this question has been investigated in only a limited set of intercropping systems (Waldron et al., 2017). The GMA/SMA approach is feasible to integrate into the later stages of a breeding pipeline, but in order to make rapid progress, intercrop breeding needs to be applied recurrently and in earlier breeding stages (Wright, 1985; Hill, 1996; Sampoux et al., 2020). In early breeding stages, the massive number and diversity of possible intercrop combinations between any two species are intractable with the full-factorial designs necessitated by the GMA/SMA approach.

Genomic and phenomic technologies potentially make early stage and rapid recurrent intercrop selection more feasible by enabling strategic rather than complete sampling of intercrop combinations and the use of partial-factorial designs. The use of genomics and especially genome-wide marker data to enhance breeding decisions is becoming pervasive across crop and livestock breeding (Butruille et al., 2015; Bernardo, 2016; Hickey et al., 2017; Georges et al., 2019; VanRaden, 2020). The process of choosing new parents or advancing new candidate cultivars on the basis of genomics-enabled predictions (GP) of their performance is known as genomic selection (GS; Meuwissen et al., 2001). GS has previously been suggested for improving mixture performance but has not yet been applied to do so (Annicchiarico et al., 2019; Bančič et al., 2021; Bourke et al., 2021; Wolfe et al., 2021).

In this special issue, two simulation studies (Bančič et al., 2021; Haug et al., 2021) and a perspective article (Wolfe et al., 2021) collectively highlight the advantage GS has to offer intercrop breeding. Haug et al. (2021) showed a clear advantage of partial-factorial designs even without using genomic information. Bančič et al. (2021) simulated several designs for using GS and sparse sampling in a two-species recurrent selection program all of which outperformed phenotypic selection. Bančič et al. (2021) modeled performance in pure vs. mixed stands as genetically correlated traits enabling breeding designs to be flexible and use a combination of mixed and pure plot trials, a feature likely to facilitate integrating intercrop breeding within established monoculture programs. Wolfe et al. (2021) point out that from a quantitative genetics perspective, the phenotype of any individual is the result of its response to an environment that is partially (or largely) determined by the other individuals present, both current and past.

The advantages of intercropping like yield stability and improved soil condition (i.e., ecosystem services) are observable primarily over multiple seasons and locations and occur because of multiple interacting species. For this reason, joint-selection approaches enabled by genomic prediction and sparse designs are needed. Genomic prediction approaches that model GxE and genome-by-genome (GxG) interactions (Burgueño et al., 2012; Cuevas et al., 2016) should therefore be adaptable to enable intercrop-level selection in either a trait-blind or trait-informed way.

As in breeding programs focused on monoculture systems, breeding material developed for intercropping systems will go through the process of variety testing and release. Variety trials should be undertaken in intercropping systems, but monoculture trials may be useful as well, depending on the cropping system and breeding context. Variety release may have added complications when dealing with multiple species, especially if intercrop compatibility is highly variety-specific, and could necessitate unconventional variety release arrangements.

Optimal methods for breeding for intercropping have been described in the literature, and some studies have validated specific breeding methods for intercropping systems. However, there is a lack of literature bridging the scales between the conceptual and the specific to describe the design of breeding pipelines in the context of specific intercropping systems. Below, we describe three intercrop breeding systems in some detail. We hope readers will draw parallels between and see differences among these cases. Our nuanced and more specific understanding of each system will in turn inform design and implementation of future intercrop breeding programs.

## Forage Mixtures

Forages are frequently grown in grass-legume mixtures (Riday and Brummer, 2014). In alfalfa (*Medicago sativa*), the most widely grown forage crop, planting practices vary by region, with mixtures more common in the northeast and upper Midwestern United States (Undersander et al., 2011). White clover (*Trifolium repens*) is grown almost exclusively in mixed stands (Riday and Brummer, 2014). In general, legumes are weaker competitors compared to grasses, with the exception of alfalfa which often dominates mixtures when included as the legume component (Haynes, 1980; Zannone et al., 1986; Jones et al., 1989; Annicchiarico and Proietti, 2010; Brophy et al., 2017; Maamouri et al., 2017). Estimates of the optimum legume percentage for maximum dry matter, protein, and animal production range between 20 and 50% (Thomas, 1992). However, environmental conditions (e.g., temperature, moisture, soil pH, and fertility) and management (e.g., harvest height and timing) can affect the proportion of each component (Jungers et al., 2019).

Grass-legume forage mixtures can provide important production benefits. Numerous studies have found forage mixtures to provide increased yield relative to grass or legume monocultures, and also greater yield stability over the growth season and/or over a multi-year period (Zannone et al., 1986; Menchaca and Connolly, 1990; Annicchiarico and Piano, 1994; Sleugh et al., 2000; Malhi et al., 2002; Frankow-Lindberg et al., 2009; Picasso et al., 2011; McElroy et al., 2012; Papadopoulos et al., 2012; Finn et al., 2013; Sanderson et al., 2013; Bélanger et al., 2014; Sturludóttir et al., 2014; Tracy et al., 2016). Additional documented benefits include improved forage quality (Sleugh et al., 2000; Malhi et al., 2002; Bélanger et al., 2014), reduced pest pressure (Lamp, 1991; Roda et al., 1996; Picasso et al., 2008; Frankow-Lindberg et al., 2009; Drenovsky and James, 2010; Sanderson et al., 2012, 2013; Finn et al., 2013; Bélanger et al., 2014; Sturludóttir et al., 2014), and reduced input needs (Zemenchik et al., 2001; Malhi et al., 2002; Nyfeler et al., 2009; Rasmussen et al., 2012; Frankow-Lindberg and Dahlin, 2013; Crème et al., 2016). Including grass species in forage mixtures have been shown to increase fiber digestibility, reduce bloating (Majak et al., 2003; Veira et al., 2010), and improve stand persistence (Sleugh et al., 2000) while the legume component fixes nitrogen (Thomas, 1992; Carlsson and Huss-Danell, 2003; Temperton et al., 2007; Nyfeler et al., 2011) and improves nutritive value (Barnett and Posler, 1983; Sleugh et al., 2000).

As mentioned above, forage mixtures often display asymmetrical competition, with the legume as the weaker competitor in most cases (Haynes, 1980; Annicchiarico and Proietti, 2010; Bybee-Finley et al., 2016; Brophy et al., 2017). This can be problematic if the legume proportion in the mixture drops to lower levels since the ecosystem services provided (e.g., N fixation) will be reduced as well (Thomas, 1992). The competitive dynamics between component species also change over time (Zannone et al., 1986; Chamblee and Collins, 1988; Marquard et al., 2009; Picasso et al., 2011; Baxter et al., 2017); in some respects, this is beneficial. For example, within a given season, temporal niche differentiation may allow one component to maximize its growth while the other is dormant (Zannone et al., 1986; Dong et al., 2018). Over a multi-year period, some species may experience reductions in plant populations and/or yield, while others show yield increase, e.g., due to compensation (Picasso et al., 2011). This can allow greater yield stability and persistence overall. However, forage quality parameters may be less stable and predictable compared to forages grown in monoculture (Grieder et al., 2021), which can bring added management complexity for producers.

Breeding forages for performance in mixtures has received more attention than many other temperate intercropping systems (Annicchiarico et al., 2019). Among forages, white clover-grass mixtures have a longer history of research and breeding, since white clover is grown predominantly in mixtures (Dijkstra and De Vos, 1972; Hill, 1990). Other forage legumes, including alfalfa, red clover (*Trifolium pratense*), and birdsfoot trefoil (*Lotus corniculatus*) have received less attention in terms of breeding specifically for mixture systems (Riday and Brummer, 2014).

The importance of breeding for forage mixtures has been established across multiple species by screening for variation in mixture performance and GxM interactions. Studies across multiple species have established variation for performance in mixtures (Atwood and Garber, 1942; Annicchiarico, 2003; Maamouri et al., 2015). The significance of GxM interactions among mixtures and pure stands varies across studies. When planting alfalfa with or without a grass companion, Zannone et al. (1986) found the best mixtures were composed of the highest-yielding genotypes when planted in monoculture, indicating a lack of GxM interaction. However, other studies in white clover (Dijkstra and De Vos, 1972; Caradus et al., 1989; Annicchiarico, 2003), alfalfa (Maamouri et al., 2017) and orchardgrass (*Dactylis glomerata*; Xie et al., 2014) have found low correlation or significant GxM interaction between mixtures and monocultures. In general, less work has focused on genetic variation and GxM interaction in grasses than in legumes (Waldron et al., 2017). The significance of GxM also likely varies with the competitiveness of the companion species and the diversity of breeding material included in a given trial (Hill and Michaelson-Yeates, 1987; Annicchiarico and Piano, 1994; Grieder et al., 2021).

To implement a trait-informed breeding approach, studies have screened for candidate traits that impact performance in mixtures, and in some cases used these traits as selection criteria in breeding programs. Haynes (1980) describes a wide range of traits that impact competitive ability in grass-legume forage mixtures, including both physiological traits (e.g., symbiosis with microbes, growth rate, and phenology, light requirement, and water use) and morphological traits (e.g., growth habit, foliage architecture, and root morphology). Most traits specifically examined in an experimental setting fall into the latter category. Many studies have evaluated morphologically divergent material not selected in the same environment (e.g., Evans et al., 1985; Turkington, 1989; Elgersma and Schlepers, 1997), which limits conclusions that can be drawn due to confounding variables (Riday and Brummer, 2014). In white clover, traits including leaf size, stolon density, and petiole elongation and plasticity have been found to be associated with performance in mixtures (Atwood and Garber, 1942; Dijkstra and De Vos, 1972; Annicchiarico and Piano, 1994; Annicchiarico, 2003). Martin and Field (1984) found in a study of white clover and perennial ryegrass (*Lolium perenne*) that both shoot and root characteristics played a role in competitive dynamics but that their relative importance shifted over time. Zarrough et al. (1983) found that tall fescue (*Festuca arundinacea*) genotypes with low-density, high-yielding tillers allowed for greater contributions of birdsfoot trefoil in a mixed stand. Short and Carlson (1989) successfully improved orchardgrass compatibility with birdsfoot trefoil by selecting for traits including canopy height, tillering, and maturity. In alfalfa, Maamouri et al. (2017) identified internode length, shoot number, leaf size, and growth habit as key traits mediating competitive ability. Across species, most of the examined traits are related to competition and niche differentiation (e.g., access to light, nutrients, and other resources) rather than facilitation.

Trait-blind approaches have also been used when selecting forages for mixture systems. Forage breeding nurseries are frequently planted in a spaced-plant arrangement for efficient data collection, distinguishing among individual plant genotypes, and increasing environmental uniformity (Casler and van Santen, 2010). However, such arrangements also eliminate competition both within and among species, and alternate arrangements may be more appropriate when selecting for intercropping systems. Riday and Brummer (2014) selected birdsfoot trefoil with and without a grass companion and found improved vigor and persistence among those selected with the grass. Forage legume breeding programs also commonly plant grasses for weed suppression purposes in space planted nurseries, with the additional benefit of selecting for performance in grass-legume mixtures (Riday and Brummer, 2014). Creeping red fescue (*Festuca rubra*) is often used since it is relatively prostrate and allows for easier viewing of space plants. However, this growth habit is quite different from that of forage grasses commonly planted with alfalfa and other legumes. The operating assumption is that creeping red fescue is an adequate proxy for other forage grasses (i.e., the effect of specific combining ability is small). Few studies have evaluated this assumption, but Grieder et al. (2021) tested red and tall fescues including both forage- and turf-types and found high phenotypic correlation between alfalfa cultivars planted across cultivation systems.

Although the efficiency of different selection schemes varies by system, previous research shows that direct selection in mixtures is most efficient when selecting forages for mixed systems. Rowe and Brink (1993) calculated predicted response to selection of white clover when planted in mixture and monoculture and found that planting in mixture would be 12–31% more effective when mixtures are the target cropping system. Where Annicchiarico (2003) compared a trait-informed approach (planted in pure stand) with two trait-blind approaches (direct selection in mixtures and selection in pure stand for biomass) in white clover and found that direct selection in a mixture was most efficient, followed by the trait-informed approach. Likewise, Waldron et al. (2017) compared selection of tall fescue in monoculture and in mixture and found direct selection in mixture to be more efficient.

Previous studies have found some specificity in the performance of forage mixtures depending on partner species. Rumbaugh and Pendery (1991) planted alfalfa clones with five associated forage species and found a significant genotype × species interaction, indicating the importance of SMA in this case. When comparing white clover performance in monoculture and in mixture with several grass species, Annicchiarico and Piano (1994) found variability of clover genotype performance to be driven by the competitiveness of the grass companion, indicating the possibility of identifying species groupings or companion “testers” based on vigor or other key traits. Given the large number of potential species pairings in forage mixtures, identification of tester species would be extremely valuable.

Over the long history of selecting forages for performance in mixtures, the bulk of breeding efforts for forage mixtures has been focused on improving the competitive ability of the less competitive species. Simultaneous selection of both grass and legume mixture components has been minimal, and we were also unable to identify programs using genomic selection as a tool in forage mixture programs. Developing forage breeding programs utilizing these approaches could increase efficiency and improve forage mixture yields.

Recognizing the ecosystem advantages of perennial forages and forage mixtures compared to annual grain production systems, efforts have been initiated to develop dual-purpose perennial grain and forage crops that produce both human edible grain and valuable forage. Of these, intermediate wheatgrass (IWG), a perennial cool-season forage grass (Ogle et al., 2011), is one of the most promising. Efforts to domesticate IWG as a dual-purpose perennial grain-forage crop were initiated in the 1980s because it produces higher seed yields relative to most perennial grasses (Knowles, 1977; Lee et al., 2009) and has edible seeds, synchronous seed maturity, low shattering, and disease resistance (Wagoner, 1989, 1990). Continuous IWG breeding and domestication efforts have been underway since 2001 and it is now the nation’s first commercially available perennial grain crop (i.e., Kernza®; DeHaan et al., 2018; Bajgain et al., 2019).

**Figure 3:**
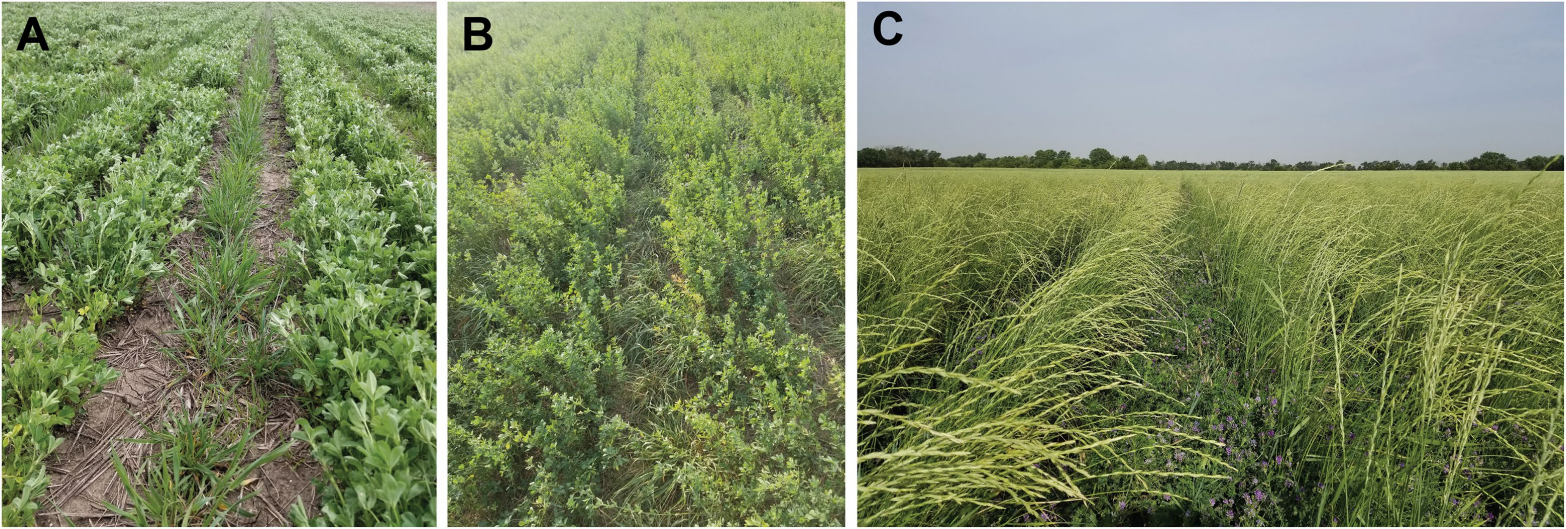
Intermediate wheatgrass (IWG) intercropped with alfalfa for dual-purpose Kernza® perennial grain and forage production in a field near Canton, KS. The IWG is planted on 30-in rows with two rows of alfalfa (10 in apart) between each pair of IWG rows. Available spring (**A**, April 6, 2020) and fall (**B**, October 14, 2020) forage and ripening Kernza® perennial grain (**C**, June 24, 2020) are shown.

Grazing IWG in the late fall, winter or early spring, much like dual-purpose management of winter wheat is common in the High Plains (Lollato et al., 2017), could increase the profitability and early adoption of IWG for Kernza® perennial grain production (Jungers et al., 2017; Ryan et al., 2018; Pugliese et al., 2019). However, the majority of the IWG annual biomass production, which can exceed 10 Mg ha^−1^, is low quality straw (crude protein <60 g kg^−1^) remaining after the grain is harvested (Favre et al., 2019). Intercropping IWG with alfalfa could improve the forage yield, quality, and seasonal distribution compared to IWG monocultures (Barnett and Posler, 1983; Sleugh et al., 2000; Aponte et al., 2019; Favre et al., 2019). Intercropping with alfalfa has also been identified as a potential strategy to meet IWG nitrogen (N) demands in perennial grain systems (Crews et al., 2016) and maintain stable IWG grain yields across years (Tautges et al., 2018; Figure 3).

Designing and breeding for IWG-alfalfa dual-purpose forage and grain production will likely be very different than for mixtures managed solely for forage. For example, in dual-purpose systems, IWG grain becomes the primary breeding target and forage yield is a secondary target, which has implications for selection decisions. IWG grain breeders are focused on improving seed size, seeds per head, and percent naked seed, rather than forage yield or quality-related traits (DeHaan et al., 2018). Breeding for IWG-alfalfa intercropping systems is in the initial stages of defining the problem space (cropping system goals) for the species and assessing variation in commercial alfalfa varieties for impact on IWG grain yield and quality. Potential traits of interest include altered alfalfa growth habit (e.g., decumbent vs. prostrate growth), temporal distribution of growth (i.e., fall dormancy), or N-fixation potential to improve IWG-alfalfa complementarity, IWG-alfalfa forage yields and quality, and efficiency of IWG grain harvest in IWG-alfalfa dual-purpose systems. Regardless of breeding goals and trait targets, there is evidence that genotype × management interactions exist in this cropping system, at least for the IWG, that are better observed in sward than in spaced plant breeding nurseries (Mortenson et al., 2019).

## Perennial Groundcover Systems

**Figure 4:**
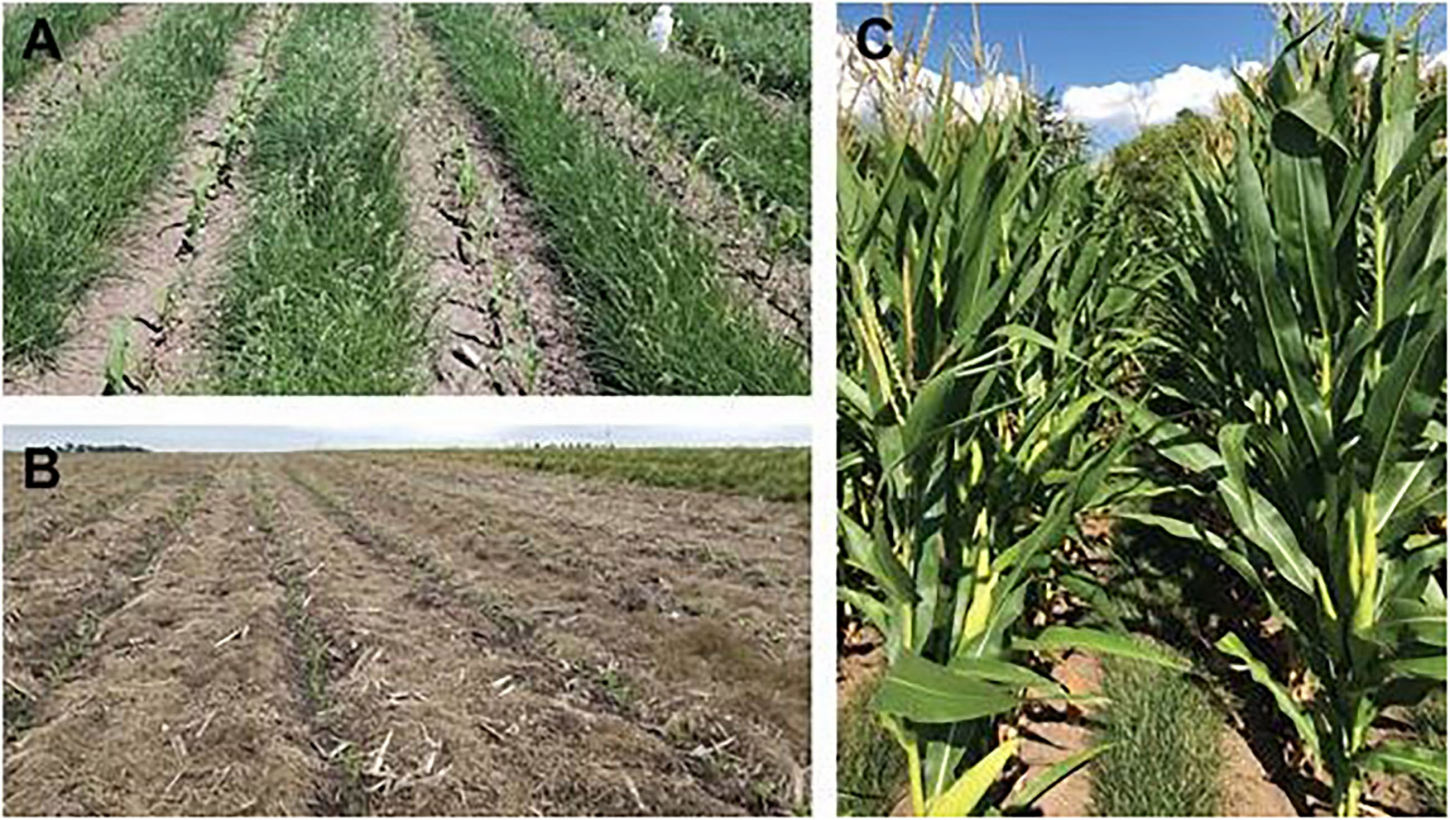
Examples of maize intercropped with turfgrass perennial groundcovers (PGC). **(A)** Maize intercropped with strip-tilled but not chemically suppressed Kentucky bluegrass PGC. **(B)** Maize intercropped with a creeping red fescue PGC that has been chemically suppressed. Chemical suppression reduces the likelihood that the maize undergoes a shade avoidance response (SAR), which results in yield loss. **(C)** Maize intercropped with Kentucky bluegrass on August 2, 2020 in Ames, IA, United States.

Perennial groundcover systems are an emerging form of intercropping which pairs high-yielding row crops (e.g., maize, soy, cotton, and sorghum) with ecologically complementary PGCs (e.g., turfgrasses and clovers; Figure 4) to achieve productivity and natural resource conservation outcomes in the same field (Moore et al., 2019). The primary role of the groundcover is to provide continuous soil cover that radically reduces soil displacement from within crop fields and delivery to surface waters (Grabber and Jokela, 2013; Schlautman et al., 2021). It is critical that in this role, the PGC species do not interfere with the cash crop, whose primary role is maximum productivity and economic return (Flynn et al., 2013; Sanders et al., 2017).

Nearly all PGC research has been conducted using cash crop and PGC species and cultivars that were bred and adapted for other purposes and management practices (Moore et al., 2019). Researchers have found that creating spatial and temporal niche differentiation between the cash crop and PGC are critical to reduce interspecific competition and avoid reductions in cash-crop productivity (Bartel et al., 2020). Without access to PGC-adapted germplasm, adequate spatial and temporal differentiation have mainly been accomplished through management using mechanical (i.e., strip-tillage) and chemical (i.e., banded applications of contact herbicides) suppression of the PGC during key periods of the cropping season—at or just before planting and during the cash-crop critical weed-free period (Martin et al., 1999; Wiggans et al., 2012a; Bartel et al., 2017; Alexander et al., 2019). However, inter- and intraspecific variation in compatibility has been observed in screens of candidate PGCs, suggesting that spatial and temporal niche differentiation between component species in PGC systems can and should be improved through breeding (Flynn et al., 2013; Verret et al., 2017).

Because multiple cash crop species will be planted in rotation in PGC systems, PGCs generally must be compatible with multiple row-crop species and varieties (i.e., have high GMA) to fit within the desired crop rotation system. Examination of the more successful PGC candidate species reveals some shared common traits: low-growing growth habit, moderate to excellent shade tolerance, excellent winter hardiness, and shallow fibrous roots (Table 1). Together, these traits allow PGC to occupy spatial and temporal niches that do not overlap significantly with cash crops in corn-soybean rotations (Flynn et al., 2013; Bartel et al., 2017; Moore et al., 2019; Schlautman et al., 2021).

**Table 1:** Turfgrass and maize ideotypes in monoculture and intercropping systems.

	Crop and cropping system		
	Turfgrass		Maize
	Monoculture	Intercropped	Intercropped
**Trait category**			
Agronomic	Winter hardiness, dark green color, fine leaf texture, and high shoot density.	*Low input needs, winter hardiness.*	Early-season vigor, cold tolerance for non-tilled, lower temperature soils.
Phenology	Reduced summer dormancy to maintain year-round green color.	Early maturing, summer dormancy to reduce maize SAR.	
Architecture	Deep rooting, short stature, and reduced growth to minimize mowing.	Short, prostrate growth habit, and shallow, fibrous root systems for reduced above- and below-ground competition.	Deep rooting system that extends beyond the PGC root system.
Abiotic	Drought and heat tolerance for year-round persistence.	Shade tolerance to persist under the maize canopy, enhanced wheel traffic tolerance.	Drought tolerance, reduced SAR under altered red/far red light conditions.
Biotic	Ability to host fitness-enhancing endophytes.	Biological nitrification inhibitors to reduce N-loss, AMF.	

In each trait category, contrasting turfgrass breeding objectives are highlighted across the cropping systems. Characteristics of the intercropped maize ideotype are also highlighted.

Cool-season grasses, and some cool-season legumes [e.g., kura clover (*Trifolium ambiguum* M. Bieb) and white clover (*T. repens* L.)] possess some or all of these desirable traits. The group of approximately 20 species cool-season grasses that possess culmless stems, making them mowing- or grazing-tolerant, commonly referred to as turfgrasses, appear especially well-suited as PGC in corn-soybean rotations (Hyder, 1972; Flynn et al., 2013). Turfgrasses have shallow fibrous root systems and they thrive in cool-moist climates (Beard, 1972). They have the C3 photosynthetic pathway, with an optimum growth temperature between 15.5 and 23.9°C (Beard, 1972), which is much cooler than the optimum growth temperatures for maize and soybean: around 30°C for vegetative growth (Hesketh et al., 1973; Sánchez et al., 2014) and 26°C for anthesis (Boote et al., 2018). In studies using Kentucky bluegrass (*Poa pratensis* L.) and red fescue (*F. rubra* L.) as PGC with maize, minimal or no reduction in grain yield was observed when the turfgrasses were chemically suppressed during maize establishment, and increases in soil water content were observed, potentially because the PGC functioned as a living mulch (Wiggans et al., 2012a,b).

**Figure 5:**
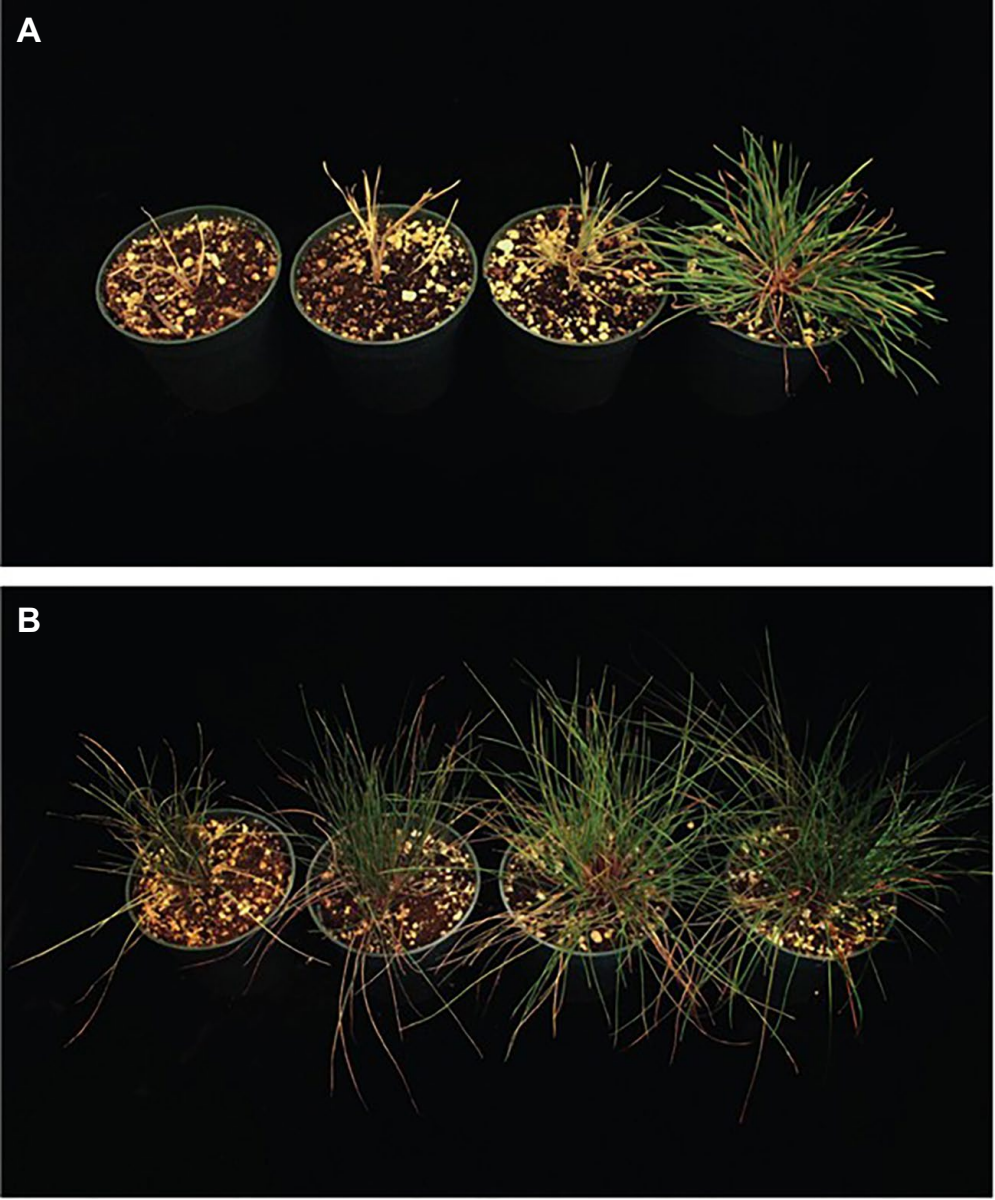
**(A)** Summer dormancy response of sandberg bluegrass (*Poa secunda* J. Presl.) accession PI 232348 to various photothermal combinations representative of Ames, IA, United States. **(B)** Non-summer dormant red fescue (*Festuca rubra* L.), cv. “Audubon” under identical photothermal conditions. Summer dormant PGCs could reduce competition with cash-row crops (e.g., maize) during the growing season.

Summer dormancy, which is strongly expressed in bulbous bluegrass (*Poa bulbosa* L.) and a few other *Poa* species, can further reduce the overlap period to be nearly non-existent. Induction and release of summer dormancy in *P. bulbosa* are controlled by photoperiod and to a lesser degree by temperature (Ofir and Kigel, 1999); therefore, its expression is strongly predictable (Figure 5). Summer dormancy can occur in Kentucky bluegrass but is likely an ecophysiological response to unfavorable environmental conditions, most likely low soil moisture, and is therefore not as reliable (Ervin and Koski, 1998; Suplick-Ploense and Qian, 2005). Summer dormancy is a key PGC trait because it has the potential to make chemical suppression of PGC unnecessary, reducing labor and cost and fitting well within both conventional and organic systems.

Perennial groundcover management undoubtedly affects the microclimate for maize or other cash crops compared to conventional management by altering the quality of the light, soil temperature, soil moisture, soil structure, and the biotic complexity of the agroecosystem (Wiggans et al., 2012b; Flynn et al., 2013; Banik et al., 2020). A few studies have demonstrated genetic variation in maize hybrid performance under PGC management (Ziyomo et al., 2013; Bowden, 2014); however, the relative importance of genotype × management (GxM) interactions to maize hybrid performance remains unknown. If crossover GxM interactions (i.e., different maize hybrids are optimal under the two management conditions) exist, then establishing dedicated breeding programs for PGC-adapted maize is advisable. If not, then the elite, locally adapted germplasm from existing breeding programs can be utilized for PGC systems.

Many potential breeding targets exist for maize adaptation to PGC management including increased tolerance to shade competition and other abiotic stresses (e.g., cold soil temperatures) as seedlings, tolerance of drought conditions as mature plants, and perhaps tolerance to unknown pests for which the PGC may provide new habitats (Berti et al., 2021). Early indications suggest that minimizing the shade avoidance response (SAR) in maize will be critical to achieving yields under PGC management that are equivalent or better than those under conventional management (Moore et al., 2019). A green PGC, even if low-growing and minimally competitive, can alter the spectrum of reflected light received by maize leaves, causing the maize to perceive potential competitors and triggering a SAR (Rajcan et al., 2004), i.e., a cascade of physiological and morphological changes that can cause irreversible crop yield loss when it occurs during the crop’s critical weed-free period (Bosnic and Swanton, 1997). While chemical suppression (which desiccates the PGC) or summer dormant PGC (whose leaves desiccate naturally) can reduce SAR, we expect that the maize SAR in PGC management could be mitigated through maize breeding. Population density insensitive maize hybrids provide evidence that maize can be, and indeed already has been, bred to tolerate intraspecific competition (Messina et al., 2021). Although still unknown, some of the same physiological mechanisms may allow maize to tolerate or fail to perceive interspecific competition in PGC management.

Cultivar development for PGC-based cropping systems is lacking. Cultivars and accessions that have been evaluated for their suitability as PGC are either wild collections or cultivars from turfgrass and forage grass breeding programs. Traits desirable for turfgrass are improved esthetic quality, which is a complex trait consisting of a number of component traits, such as shoot density, leaf color, and texture while traits desirable for forage grasses are high biomass yield and better nutritional quality. These traits are not inherently in conflict with traits for PGC except summer persistence, also a complex trait that is highly desirable for turfgrass cultivars and most forage grass cultivars but may be of less importance to PGC. Despite a relatively short history in turfgrass breeding, a large number of turfgrass cultivars have been released for major turf species including Kentucky bluegrass, Tall fescue (*F. arundinaceae* Schreb.), perennial ryegrass (*L. perenne* L.), and red fescue (National Turfgrass Evaluation Program, http://ntep.org). There are also numerous wild collections for major cool-season grasses and legumes maintained at USDA GRIN,^1^ most of which have not been well characterized and serve as an untapped resource for developing dedicated PGC germplasm. For species such as Kentucky bluegrass in which apomixis is the predominant reproductive mode or for species that reproduce by vivipary as in *P. bulbosa*, unimproved wild accessions may be directly deployed as PGC following field evaluation and seed increase. It is well known that abundant variation exists among cultivars of major cool-season grasses. For example, cultivars of Kentucky bluegrass vary so greatly that there are at least 16 groups of cultivars that each differs in morphology and development patterns from others (Honig et al., 2012). Screening of commercially available cultivars for their “mixing ability” with row crops is the most cost-effective strategy at this point to further refine the ideotype for PGC and facilitate trait-informed selection in the future.

The availability of compatible PGC cultivars that maintain adequate ground coverage without causing yield reduction to row crops is critically important to the success of PGC-based cropping systems. No dedicated PGC cultivars are currently available and the need for developing such cultivars is clearly present. Unlike selection and cultivar development for monoculture which deals with intraspecific interactions (typically among highly related plants within the same species), selection for PGC for intercropping has to consider the unique interspecific interactions. The inter-row space where PGC is grown is a unique microenvironment where air and soil temperatures, air and soil moisture, and light quality all differ from that of monoculture. It is therefore important that selection for superior genotypes is done in such an environment. While the near-term goal for breeding PGC is to identify or develop cultivars that provide adequate ground coverage without reducing yield of the row crop, future breeding needs to develop value-added traits such as the ability to inhibit nitrification (biological nitrification inhibition, BNI; Subbarao et al., 2007, 2009) or the enhanced capability of arbuscular mycorrhiza (AM) colonization. BNI can reduce N leaching and improve N use efficiency and has been reported in a number of grass species including perennial ryegrass (Moore and Waid, 1971; Subbarao et al., 2021). Symbiont AM can help plants capture nutrients such as phosphorus from soil (Deguchi et al., 2017). PGC cultivars with value-added traits should facilitate adoption of the PGC-Crop system.

## Intercropping Soybeans With Winter Oilseeds

Numerous legume-oilseed intercropping systems have been developed, and some have shown significant potential for commercial potential (e.g., canola-pea intercropping) and advantages in terms of yield and nutrient-use efficiency (Dowling et al., 2021). In this case study, we focus on the development of novel intercropping systems including soybeans and winter oilseed crops. Winter oilseeds are being incorporated into existing cropping systems as an alternative to traditional winter annual cover crops. Like cover crops, they can provide environmental benefits (e.g., winter soil protection) and can also be harvested as a cash crop. Intercropping with winter oilseeds has become feasible with recent advances in domesticating cold hardy brassicas (Frels et al., 2019; Marks et al., 2021). In regions with harsh winter conditions, field pennycress (*Thlaspi arvense*) and winter camelina (*Camelina sativa*) offer suitable options (Cubins et al., 2019; Zhang and Auer, 2019). For regions that experience a milder winter, carinata (*Brassica carinata*) serves as a cool-season alternative (Gasol et al., 2007). Members of the Brasicacea family are particularly suitable for this cropping system due to their cold tolerance (Warwick, 2011; Song et al., 2020). Furthermore, this adaptation for winter growth can capitalize on the observed increases in winter temperatures for the Northern Hemisphere (McCabe and Wolock, 2010).

**Figure 6:**
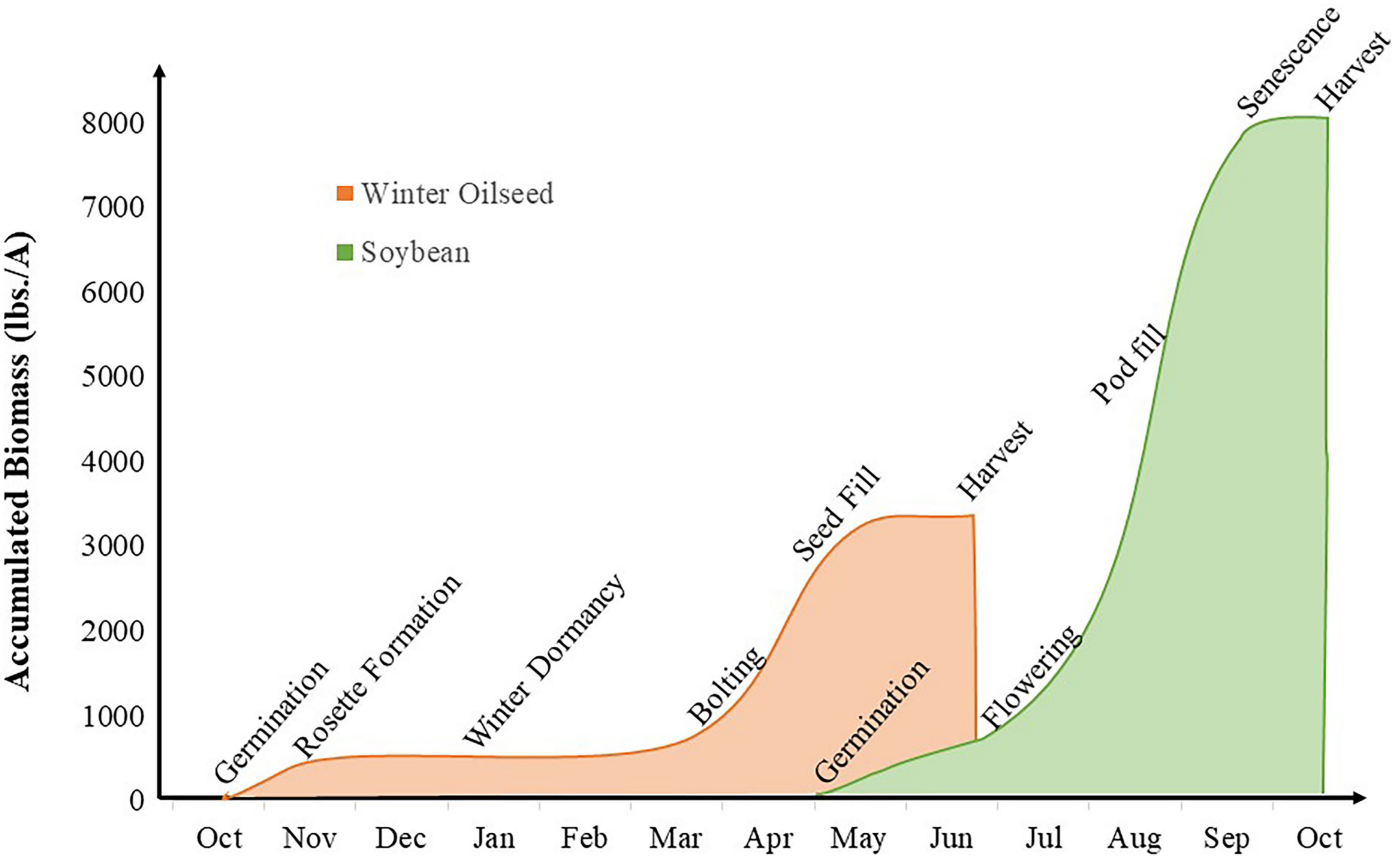
Intercropping interaction window for winter oilseed intercropping systems. The orange curve represents biomass production and key growth stages in winter oilseed crop production and the green curve represents biomass production and key growth stages in soybean production.

In intercropping systems that include winter oilseeds, yield of both winter and summer crops are considered primary breeding goals. To a greater degree than in other systems previously discussed, these systems accomplish niche differentiation through temporal separation of the component crops (Brooker et al., 2015); there are extended periods in which a single species is growing in the field and narrower windows of overlap among the component crops. Winter annuals are established in the fall and harvested in early summer. In intercropping systems, the winter annuals are interplanted with summer annual row crops such as corn or soybean prior to seed formation (Figure 6). This strategy reduces the fallow period between crops, provides ecological benefits such as pollen for early-season pollinators and reduced leaching of nitrogen into groundwater sources (Weyers et al., 2019; Forcella et al., 2021), and produces harvestable yield from both cropping system components.

**Figure 7:**
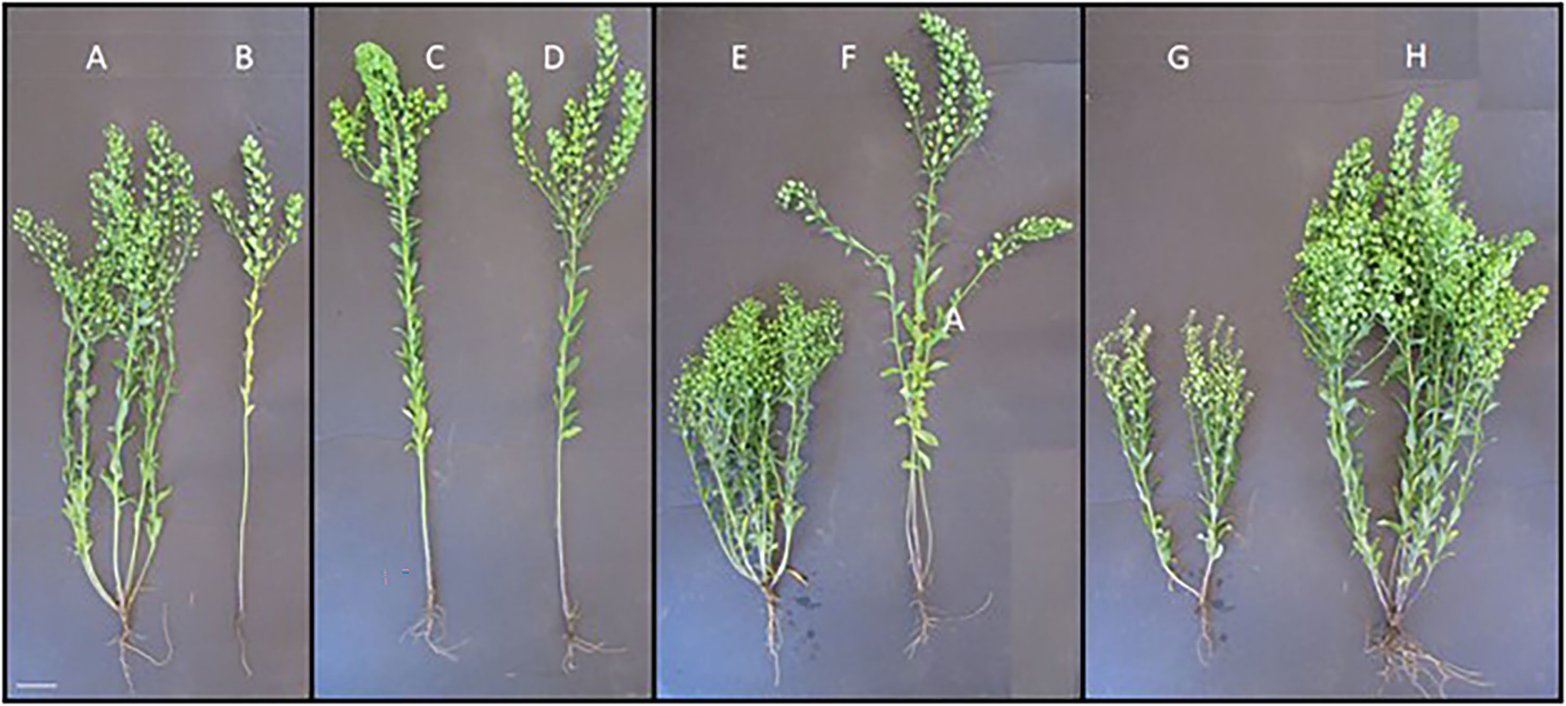
Trait variation in pennycress. **(A)** Wild type, **(B)** non-tillering, **(C)** Fasciated, **(D)** wild type, **(E)** dwarfing, **(F)** wild type, **(G)** glucosinolate null, and **(H)** wild type.

As most breeding programs for these winter annuals are less than a decade old, their focus has mainly been on key domestication traits (Chopra et al., 2020). Advances have also been made for heritable variations in plant morphology in the University of Minnesota’s pennycress breeding program (Figure 7). Active breeding for intercropping systems has only recently been initiated, and there is a need to define an ideotype for intercropped winter oilseeds to facilitate the breeding of cultivars specifically adapted to intercropping to maximize yield. Some key differences in breeding objectives between monoculture and intercropping systems are likely to be important (Table 2).

**Table 2:** Winter oilseed and soybean ideotypes in monoculture and intercropping systems.

	Crop and cropping system			
	Winter oilseed		Soybean	
	Monoculture	Intercropped	Monoculture	Intercropped
**Trait category**				
Agronomic	Reduced shattering to maximize harvestable yield.	*Reduced shattering to maximize harvestable yield and prevent late-season competition.*	Early-season vigor, cold tolerance.	*Early-season vigor, cold tolerance for non-tilled, lower temperature soils.*
Phenology	Late maturity to maximize seed yield.	Early maturity for early harvest to minimize competition window.	Early flowering and long reproductive period to maximize yield.	Late flowering so reproductive period does not overlap with intercropping competition.
Architecture	Reduced height for reduced lodging. Increased tillering to maximize yield.	Increased height of first silique for harvestability above soybean canopy. Reduced tillers to reduce competitive impact on soybean.	Rapid canopy closure through enhanced branching. Deep rooting.	*Rapid canopy closure through enhanced branching. Deep rooting*.
Abiotic	Nutrient-use efficiency.	*Nutrient-use efficiency for growth in competition with soybean.*	Drought tolerance.	*Drought tolerance, especially early-season in moisture-depleted soils.* Shade tolerance.
Biotic	High glucosinolates to suppress weed pressure.	Low glucosinolates to reduce allelopathic effects on soybean.		Tolerance to allelopathy.

In each trait category, contrasting breeding objectives are highlighted across the cropping systems. Italic text indicates that the breeding objective is of greater importance in one cropping system than the other.

Soybeans (*Glycine max*) offer a compatible and plastic option as a relay crop in a winter oilseed production system (Hussain et al., 2020). In the United States, soybeans are commonly double cropped with winter wheat, especially in parts of the Mid-South Region (Chan et al., 1980; Buehring et al., 1990; Wallace et al., 1992). Soybeans are cultivated on a global scale with greater than 120 million Ha harvested in 2019 [Food and Agriculture Organization (FAO), 2019]. With such a large distribution, there are a plethora of intercropping studies between soybeans and various component species (sugarcane: Li et al., 2013; cassava: Tsay et al., 1988; sunflower: Saudy and El-Metwally, 2009; maize: Fan et al., 2020; and wheat: Li et al., 2001). In a winter annual oilseed relay system, soybean yield is highly dependent on environmental conditions with observed reductions in yield ranging from 0 to 47% (Hoerning et al., 2020). Davis and Woolley (1993) also note that the genotype-by-cropping system interaction is more important for the understory crop (in this case the soybean). Negative effects on soybean yield may be due to a range of stressors including direct competition for resources, increased pest pressure, and allelopathy.

Direct competition in this system occurs on a shorter time frame than other intercropping systems, yet the effects on juvenile soybean can have long lasting consequences for yield components, including height, biomass, and pod counts (Ott et al., 2019). One of the resources that become limited is light through the rapid canopy closure in pennycress and camelina stands where 40%–70% of available photosynthetically active radiation is blocked to the underlying soybeans (Ott et al., 2019). Current research is ongoing on allowing a greater amount of light to penetrate into the canopy through employing non-tillering pennycress lines (personal communication with Ratan Chopra), and it would also be beneficial to develop shade tolerance in soybeans. Planting winter oilseeds can deplete soil moisture levels, which can result in poor germination of the summer annual crop (Gesch and Johnson, 2015). One method to ameliorate reduced soil moisture may be to choose large-seeded soybeans which have been associated with early-season vigor (Smith and Camper, 1975).

Another biotic stressor that is the consequence of cultivating two species together is the potential for one to attract pests to the later crop. For example, field pennycress has shown to be an alternative host for soybean cyst nematodes (SCN; Johnson et al., 2008; Hoerning, 2019). Soybean cultivars with strong resistance to SCN are widely available, and longer rotation schedules are also a viable option. Sclerotinia has been shown to infect pennycress under controlled conditions (Boland and Hall, 1994), but there are currently no reports of infection of cultivated pennycress in the field. However, there have been reports of field infection of Camelina (Séguin-Swartz et al., 2009). In addition, the development stage of soybean plays a significant role in pathogen dynamics. Soybeans are susceptible once they begin to flower (Peltier et al., 2012), which typically would occur after winter oilseed harvest. Nevertheless, there could be a buildup of sclerotia in the soil, making major outbreaks more likely. The pathogen dynamics in this intercropping system merit further study. Management tools that could help mitigate major outbreaks could include planting-resistant varieties, lowering planting density, using longer rotation schedules, and applying fungicides.

Allelopathy has long been a tool in cropping systems to reduce weed pressure (Putnam et al., 1983). Winter oilseeds have observed phytotoxic effects where weed biomass was suppressed up to 100% in pennycress and up to 87% in winter camelina (Hoerning et al., 2020). The only glucosinolate present in pennycress is in the form of sinigrin and the partial breakdown of sinigrin results in highly allelopathic isothiocyanates (Bialy et al., 1990; Chopra et al., 2019). Allelopathy tolerance is poorly characterized in soybeans but would be a necessity in developing varieties for a winter oilseed intercropping system.

Breeding for a winter oilseed relay system presents challenges not faced in other intercropping systems. Since winter oilseeds are still early on in the development pipeline and a market value is not yet established, it is unclear if importance should be given to maximizing the winter oilseed yield or reducing the yield penalties on soybeans. This differentiation is important in breeding for multiple traits because it has strong implications on selection indices, for example, one common approach is a base index selection where the weight of a trait is dependent on the market price (Kauffmann and Dudley, 1979). A number of breeding goals for monoculture and intercropping systems are mutually exclusive and signify the need for separate breeding pipelines. For example, earlier-flowering pennycress lines reduce the interaction window at the expense of pennycress yields, and reduced glucosinolate production corresponds to less allelopathic effects on soybeans at the expense of introducing insect and weed vulnerabilities to the pennycress (Table 2). Until the crops and markets are more established, prioritizing these breeding goals may remain difficult. Despite these challenges, the development of a winter oilseed intercropping system has shown promise in terms of increased total yield, and additional markets may benefit producers by spreading risk (Gesch et al., 2014). These advantages, combined with ecosystem services such as reduced erosion and increased pollinator support, merit further efforts in breeding and management to help expand these systems into the future.

## Discussion

While all focused on breeding for intercropping, each of these case studies is distinct in its specific objectives. In forage mixture systems, both grass and legume components are harvested together as a single product, and the breeder seeks to maximize productivity of this mixture as a whole. In the unique case of perennial grain-forage systems, the grain becomes the primary target, with forage yield as a secondary goal. By contrast, in the PGC system, only the row crop is harvested, while the PGC is grown purely for ecosystem service purposes. The breeding focus will be primarily on improving the ability of the PGC to function within the row crop context (e.g., improving survival without impacting row crop yields). Some breeding may also be conducted in the row crop for improved compatibility with the PGC (e.g., to eliminate SAR), but such breeding efforts are likely to be fruitful only if they do not impact yield. In the winter oilseed intercropping systems, both summer and winter annual crops are harvested but as two separate crops. Yield of both crops are currently treated as primary breeding goals, but the cropping system is still developing, and as the production systems and markets mature, it will be critical to determine whether breeding programs should maximize yield of one component crop at the expense of the other.

The approaches used to minimize competition and maximize complementarity are also somewhat different in each case study. In the case of both PGC and winter oilseed intercropping systems, both temporal and spatial niche differentiation are important. In PGC systems, summer dormancy (temporal) and growth habit (spatial) have been important factors driving the choice of species to include in the system and could be enhanced through breeding (Table 1). In winter oilseed systems, maturity timing and growth habit are also important selection criteria for intercropping systems (Table 2). In both systems, there is also a need to select row crop components to reduce negative interactions associated with intercropping (eliminating SAR in corn for PGC systems and reducing susceptibility to allelochemicals in soybean for winter oilseed systems). In contrast to other systems, breeding efforts for forage mixtures have seen a greater focus on spatial niche differentiation (e.g., selecting plants with compatible morphology), since component species are grown together for the entire cropping period, although facilitation (e.g., nitrogen fixation) and temporal traits (e.g., emergence and maturity timing) are also important.

Each of these cropping systems is also at a different stage in the development and implementation of a breeding pipeline for intercropping. Forage mixtures are certainly the most mature among the cropping systems discussed. Studies have been published addressing each major step along the pipeline (Figure 1) for at least some forage species and mixture combinations. Forage legume breeding also frequently takes place with a turf groundcover, mimicking a forage mixture. However, there is a need to deploy the methods described in this review in a more systematic way and to accelerate efforts to breed for mixture systems, e.g., through genomic selection and/or selection of both component species. Breeding for PGC and winter oilseed systems is comparatively much less developed. While there has been significant research focused on management of PGC systems, no breeding has taken place. Winter oilseed systems are at an early stage of development, with some species still being domesticated. In both cases, there is an opportunity to design a breeding pipeline that incorporates intercropping systems as one of its primary goals. Although nascent, breeding for intercropping systems holds great potential for improving intercropping systems and realizing the potential of this crop diversification strategy for addressing sustainability challenges.

## Author Contributions

VM contributed to conception and design of the manuscript. AL, BS, LR, MW, S-zF, and VM wrote sections of the manuscript. VM compiled and revised author contributions and wrote the first draft of the introduction and discussion. All authors contributed to the article and approved the submitted version.

## Funding

This work was in part supported by United States Department of Agriculture (USDA), National Institute of Food and Agriculture (NIFA) Awards #2019-67012-34886 and #2017-67019-26370.

## Conflict of Interest

The authors declare that the research was conducted in the absence of any commercial or financial relationships that could be construed as a potential conflict of interest.

## Publisher’s Note

All claims expressed in this article are solely those of the authors and do not necessarily represent those of their affiliated organizations, or those of the publisher, the editors and the reviewers. Any product that may be evaluated in this article, or claim that may be made by its manufacturer, is not guaranteed or endorsed by the publisher.

## Footnotes

^1^ https://npgsweb.ars-grin.gov/gringlobal/crop?id=110

## References

[ref1] Alexander J. R. Venterea R. T. Baker J. M. Coulter J. A. (2019). Kura clover living mulch: spring management effects on nitrogen. Agronomy 9:69. doi: 10.3390/agronomy9020069

[ref2] Altieri M. A. (1999). The ecological role of biodiversity in agroecosystems. Agric. Ecosyst. Environ. 74, 19–31. doi: 10.1016/S0167-8809(99)00028-6, PMID: 35124311

[ref3] Andrews D. J. Kassam A. H. (1976). “The importance of multiple cropping in increasing world food supplies,” in Multiple Cropping. eds. R. Papendick, P. Sanchez and G. Triplett (Madison, WI: American Society of Agornomy, Crop Science Society of America, and Soil Science Society of America, Inc.), 1–10.

[ref4] Anil L. Phipps R. H. Park J. Miller (1998). Temperate intercropping of cereals for forage: a review of the potential for growth and utilization with particular reference to the UK. Grass Forage Sci. 53, 301–317. doi: 10.1046/j.1365-2494.1998.00144.x

[ref5] Annicchiarico P. (2003). Breeding white clover for increased ability to compete with associated grasses. J. Agric. Sci. 140, 255–266. doi: 10.1017/S0021859603003198

[ref6] Annicchiarico P. Collins R. P. De Ron A. M. Firmat C. Litrico I. Hauggaard-Nielsen H. (2019). Do we need specific breeding for legume-based mixtures? Adv. Agron. 157, 141–215. doi: 10.1016/bs.agron.2019.04.001

[ref7] Annicchiarico P. Piano E. (1994). Interference effects in white clover genotypes grown as pure stands and binary mixtures with different grass species and varieties. Theoret. Appl. Genet. 88, 153–158. doi: 10.1007/BF00225891, PMID: 24185920

[ref8] Annicchiarico P. Proietti S. (2010). White clover selected for enhanced competitive ability widens the compatibility with grasses and favours the optimization of legume content and forage yield in mown clover-grass mixtures. Grass Forage Sci. 65, 318–324. doi: 10.1111/j.1365-2494.2010.00749.x

[ref9] Aponte A. Samarappuli D. Berti M. T. (2019). Alfalfa–grass mixtures in comparison to grass and alfalfa monocultures. Agron. J. 111, 628–638. doi: 10.2134/agronj2017.12.0753

[ref10] Arbuckle J. G. Roesch-McNally G. (2015). Cover crop adoption in Iowa: the role of perceived practice characteristics. J. Soil Water Conserv. 70, 418–429. doi: 10.2489/jswc.70.6.418

[ref11] Atlin G. Frey K. (1989). Breeding crop varieties for low-input agriculture. Am. J. Altern. Agric. 4, 53–58. doi: 10.1017/S0889189300002721

[ref12] Atwood S. S. Garber R. J. (1942). The evaluation of individual plants of white clover for yielding ability in association with bluegrass. Agron. J. 34, 1–6. doi: 10.2134/agronj1942.00021962003400010001x

[ref13] Bajgain P. Zhang X. Anderson J. A. (2019). Genome-wide association study of yield component traits in intermediate wheatgrass and implications in genomic selection and breeding. G3 9, 2429–2439. doi: 10.1534/g3.119.400073, PMID: 31147390 PMC6686922

[ref14] Bančič J. Werner C. R. Gaynor R. C. Gorjanc G. Odeny D. A. Ojulong H. F.. (2021). Modeling illustrates that genomic selection provides new opportunities for intercrop breeding. Front. Plant Sci. 12:605172. doi: 10.3389/fpls.2021.605172, PMID: 33633761 PMC7902002

[ref15] Banik C. Bartel C. A. Laird D. A. Moore K. J. Lenssen A. W. (2020). Perennial cover crop influences on soil C and N and maize productivity. Nutr. Cycl. Agroecosyst. 116, 135–150. doi: 10.1007/s10705-019-10030-3

[ref16] Bänziger M. Cooper M. (2001). Breeding for low input conditions and consequences for participatory plant breeding examples from tropical maize and wheat. Euphytica 122, 503–519. doi: 10.1023/A:1017510928038

[ref17] Barnett F. L. Posler G. L. (1983). Performance of cool-season perennial grasses in pure stands and in mixtures with legumes. Agron. J. 75, 582–586. doi: 10.2134/agronj1983.00021962007500040004x

[ref18] Barot S. Allard V. Cantarel A. Enjalbert J. Gauffreteau A. Goldringer I.. (2017). Designing mixtures of varieties for multifunctional agriculture with the help of ecology. Agron. Sustain. Dev. 37:13. doi: 10.1007/s13593-017-0418-x.covercrop

[ref19] Bartel C. A. Archontoulis S. V. Lenssen A. W. Moore K. J. Huber I. L. Laird D. A.. (2020). Modeling perennial groundcover effects on annual maize grain crop growth with the agricultural production systems sIMulator. Agron. J. 12, 1895–1910. doi: 10.1002/agj2.20108

[ref20] Bartel C. A. Banik C. Lenssen A. W. Moore K. J. Laird D. A. Archontoulis S. V.. (2017). Living mulch for sustainable maize Stover biomass harvest. Crop Sci. 57, 3273–3290. doi: 10.2135/cropsci2017.04.0232

[ref21] Baxter L. West C. Brown P. Green P. (2017). Nondestructive determination of legume content in grass-legume pastures. Crop, Forage Turfgrass Manag. 3, 1–9. doi: 10.2134/cftm2016.12.0088

[ref22] Beard J. B. (1972). Turfgrass: Science and Culture. Englewood Cliffs, New Jersey: Prentice-Hall.

[ref23] Bélanger G. Castonguay Y. Lajeunesse J. (2014). Benefits of mixing timothy with alfalfa for forage yield, nutritive value, and weed suppression in northern environments. Can. J. Plant Sci. 94, 51–60. doi: 10.4141/cjps2013-228

[ref24] Bernardo R. (2016). Bandwagons I, too, have known. Theor. Appl. Genet. 129, 2323–2332. doi: 10.1007/s00122-016-2772-5, PMID: 27681088

[ref25] Berti M. T. Cecchin A. Samarappuli D. P. Patel S. Lenssen A. W. Moore K. J.. (2021). Alfalfa established successfully in intercropping with corn in the Midwest US. Agronomy 11:1676. doi: 10.3390/agronomy11081676

[ref26] Bialy Z. Oleszek W. Lewis J. Fenwick G. R. (1990). Allelopathic potential of glucosinolates (mustard oil glycosides) and their degradation products against wheat. Plant Soil 129, 277–281. doi: 10.1007/BF00032423

[ref27] Boland G. J. Hall R. (1994). Index of plant hosts of *Sclerotinia sclerotiorum*. Can. J. Plant Pathol. 16, 93–108. doi: 10.1080/07060669409500766, PMID: 26442085

[ref28] Boote K. J. Prasad V. Allen L. H. Singh P. Jones J. W. (2018). Modeling sensitivity of grain yield to elevated temperature in the DSSAT crop models for peanut, soybean, dry bean, chickpea, sorghum, and millet. Eur. J. Agron. 100, 99–109. doi: 10.1016/j.eja.2017.09.002

[ref29] Bosnic A. C. Swanton C. J. (1997). Influence of Barnyardgrass (*Echinochloa crus-galli*) time of emergence and density on corn (*Zea mays*). Weed Sci. 45, 276–282. doi: 10.1017/S0043174500092833

[ref30] Boudreau M. A. (2013). Diseases in intercropping systems. Annu. Rev. Phytopathol. 51, 499–519. doi: 10.1146/annurev-phyto-082712-102246, PMID: 23725470

[ref31] Bourke P. M. Evers J. B. Bijma P. van Apeldoorn D. F. Smulders M. J. M. Kuyper T. W.. (2021). Breeding beyond monoculture: putting the ‘intercrop’ into crops. Front. Plant Sci. 12:734167. doi: 10.3389/fpls.2021.734167, PMID: 34868116 PMC8636715

[ref32] Bowden N. J. (2014). Adaptability potential of corn for groundcover cropping systems.

[ref33] Brooker R. W. Bennett A. E. Cong W.-F. Daniell T. J. George T. S. Hallett P. D.. (2015). Improving intercropping: a synthesis of research in agronomy, plant physiology and ecology. New Phytol. 206, 107–117. doi: 10.1111/nph.13132, PMID: 25866856

[ref34] Brophy C. Finn J. A. Lüscher A. Suter M. Kirwan L. Sebastià M.-T.. (2017). Major shifts in species’ relative abundance in grassland mixtures alongside positive effects of species diversity in yield: a continental-scale experiment. J. Ecol. 105, 1210–1222. doi: 10.1111/1365-2745.12754

[ref35] Brummer E. C. (2006). *May. Breeding for cropping systems*. in “Plant Breeding: The Arnel R. Hallauer International Symposium.” Ames, Iowa, USA: Blackwell Publishing, 97–106.

[ref36] Buehring N. W. Reginelli D. B. Blaine M. A. (1990). *Long term wheat and soybean response to an intercropping system*. in “Proceedings of the Southern Conservation Tillage Conference”, Raleigh, NC. July 16–17, 1990; 65–68.

[ref37] Burgueño J. de los Campos G. Weigel K. Crossa J. (2012). Genomic prediction of breeding values when modeling genotype × environment interaction using pedigree and dense molecular markers. Crop Sci. 52, 707–719. doi: 10.2135/cropsci2011.06.0299

[ref38] Butruille D. V. Birru F. H. Boerboom M. L. Cargill E. J. Davis D. A. Dhungana P.. (2015). “Maize breeding in the United States: views from within monsanto,” in Plant Breeding Reviews. *Vol*. 39. ed. J. Janick (John Wiley & Sons, Ltd.), 199–282.

[ref39] Bybee-Finley K. A. Mirsky S. B. Ryan M. R. (2016). Functional diversity in summer annual grass and legume intercrops in the northeastern United States. Crop Sci. 56, 2775–2790. doi: 10.2135/cropsci2016.01.0046

[ref40] Bybee-Finley K. Ryan M. R. (2018). Advancing intercropping research and practices in industrialized agricultural landscapes. Agriculture 8:80. doi: 10.3390/agriculture8060080

[ref41] Callaway R. M. (1995). Positive interactions among plants. Bot. Rev. 61, 306–349. doi: 10.1007/BF02912621, PMID: 35274744

[ref42] Caradus J. R. Mackay A. C. Bosch J. V. D. Woodfield D. R. (1989). Comparative evaluation of white clover cultivars in spaced plant and small mixed species plot trials. N. Z. J. Agric. Res. 32, 433–436. doi: 10.1080/00288233.1989.10421763

[ref43] Carlsson G. Huss-Danell K. (2003). Nitrogen fixation in perennial forage legumes in the field. Plant Soil 253, 353–372. doi: 10.1023/A:1024847017371, PMID: 27325893

[ref44] Case T. J. Gilpin M. E. (1974). Interference competition and niche theory. PNAS 71, 3073–3077. doi: 10.1073/pnas.71.8.3073 4528606 PMC388623

[ref45] Casler M. D. van Santen E. (2010). “Breeding objectives in forages,” in Fodder Crops and Amenity Grasses. eds. Boller B. Posselt U. K. Veronesi F. (New York, NY: Springer New York), 115–136.

[ref46] Chamblee D. S. Collins M. (1988). Relationships with other species in a mixture. Agron. Monogr. 29, 439–461.

[ref47] Chan L. M. Johnson R. R. Brown C. M. (1980). Relay intercropping soybeans into winter wheat and spring oats 1. Agron. J. 72, 35–39. doi: 10.2134/agronj1980.00021962007200010008x

[ref48] Cheriere T. Lorin M. Corre-Hellou G. (2020). Species choice and spatial arrangement in soybean-based intercropping: levers that drive yield and weed control. Field Crop Res. 256:107923. doi: 10.1016/j.fcr.2020.107923

[ref49] Chopra R. Folstad N. Lyons J. Ulmasov T. Gallaher C. Sullivan L.. (2019). The adaptable use of Brassica NIRS calibration equations to identify pennycress variants to facilitate the rapid domestication of a new winter oilseed crop. Ind. Crop. Prod. 128, 55–61. doi: 10.1016/j.indcrop.2018.10.079

[ref50] Chopra R. Johnson E. B. Emenecker R. Cahoon E. B. Lyons J. Kliebenstein D. J.. (2020). Identification and stacking of crucial traits required for the domestication of pennycress. Nature Food 1, 84–91. doi: 10.1038/s43016-019-0007-z

[ref51] Cong W. F. Hoffland E. Li L. Six J. Sun J. H. Bao X. G.. (2015). Intercropping enhances soil carbon and nitrogen. Glob. Chang. Biol. 21, 1715–1726. doi: 10.1111/gcb.12738, PMID: 25216023

[ref52] Connell J. H. (1983). On the prevalence and relative importance of interspecific competition: evidence from Field experiments. Am. Nat. 122, 661–696. doi: 10.1086/284165

[ref53] Connolly J. Sebastià M.-T. Kirwan L. Finn J. A. Llurba R. Suter M.. (2018). Weed suppression greatly increased by plant diversity in intensively managed grasslands: A continental-scale experiment. J. Appl. Ecol. 55, 852–862. doi: 10.1111/1365-2664.12991, PMID: 29540935 PMC5836893

[ref54] Corre-Hellou G. Fustec J. Crozat Y. (2006). Interspecific competition for soil N and its interaction with N2 fixation, leaf expansion and crop growth in pea–barley intercrops. Plant Soil 282, 195–208. doi: 10.1007/s11104-005-5777-4

[ref55] Crème A. Rumpel C. Gastal F. de la Luz Mora Gil M. Chabbi A. (2016). Effects of grasses and a legume grown in monoculture or mixture on soil organic matter and phosphorus forms. Plant Soil 402, 117–128. doi: 10.1007/s11104-015-2740-x, PMID: 33016354

[ref56] Crews T. E. Blesh J. Culman S. W. Hayes R. C. Jensen E. S. Mack M. C.. (2016). Going where no grains have gone before: From early to mid-succession. Agric. Ecosyst. Environ. 223, 223–238. doi: 10.1016/j.agee.2016.03.012

[ref57] Crews T. E. Peoples M. B. (2004). Legume versus fertilizer sources of nitrogen: ecological tradeoffs and human needs. Agric. Ecosyst. Environ. 102, 279–297. doi: 10.1016/j.agee.2003.09.018

[ref58] Cubins J. A. Wells M. S. Frels K. Ott M. A. Forcella F. Johnson G. A.. (2019). Management of pennycress as a winter annual cash cover crop. A review. Agron. Sustain. Dev. 39:46. doi: 10.1007/s13593-019-0592-0, PMID: 34990041

[ref59] Cuevas J. Crossa J. Soberanis V. Pérez-Elizalde S. Pérez-Rodríguez P. Campos G. L.. (2016). Genomic prediction of genotype × environment interaction kernel regression models. Plant Genome 9:24. doi: 10.3835/plantgenome2016.03.0024, PMID: 27902799

[ref701] Davis J. H. C. Woolley J. N. (1993). Genotypic requirement for intercropping. Field Crops Res. 34, 407–430. doi: 10.1016/0378-4290(93)90124-6

[ref60] Dawson J. C. Goldringer I. (2011). “Breeding for genetically diverse populations: variety mixtures and evolutionary populations,” in Organic Crop Breeding. eds. E. T. Lammerts van Bueren and J. R. Myers (Ames, IA: John Wiley & Sons, Ltd.), 77–98.

[ref61] Deguchi S. Uozumi S. Touno E. Uchino H. Kaneko M. Tawaraya K. (2017). White clover living mulch reduces the need for phosphorus fertilizer application to corn. Eur. J. Agron. 86, 87–92. doi: 10.1016/j.eja.2017.03.006

[ref62] DeHaan L. Christians M. Crain J. Poland J. (2018). Development and evolution of an intermediate wheatgrass domestication program. Sustain. For. 10:1499. doi: 10.3390/su10051499

[ref63] Dijkstra J. De Vos A. L. F. (1972). The evaluation of selections of white clover (*Trifolium repens* L.) inmonoculture and in mixture with grass. Euphytica 21, 432–449. doi: 10.1007/BF00039339

[ref64] Dong N. Tang M.-M. Zhang W.-P. Bao X.-G. Wang Y. Christie P.. (2018). Temporal differentiation of crop growth as one of the drivers of intercropping yield advantage. Sci. Rep. 8:3110. doi: 10.1038/s41598-018-21414-w, PMID: 29449595 PMC5814522

[ref65] Dowling A. Sadras O. V. Roberts P. Doolette A. Zhou Y. Denton M. D. (2021). Legume-oilseed intercropping in mechanised broadacre agriculture – a review. Field Crop Res. 260:107980. doi: 10.1016/j.fcr.2020.107980

[ref66] Drenovsky R. E. James J. J. (2010). Designing invasion-resistant plant communities: the role of plant functional traits. Rangelands 32, 32–37. doi: 10.2111/RANGELANDS-D-09-00002.1

[ref67] Eberle C. A. Thom M. D. Nemec K. T. Forcella F. Lundgren J. G. Gesch R. W.. (2015). Using pennycress, camelina, and canola cash cover crops to provision pollinators. Ind. Crop. Prod. 75, 20–25. doi: 10.1016/j.indcrop.2015.06.026

[ref68] Elgersma A. Schlepers H. (1997). Performance of white clover/perennial ryegrass mixtures under cutting. Grass Forage Sci. 52, 134–146. doi: 10.1111/j.1365-2494.1997.tb02344.x

[ref69] Ervin E. H. Koski A. J. (1998). Drought avoidance aspects and crop coefficients of Kentucky bluegrass and tall fescue turfs in the semiarid West. Crop Sci. 38, 788–795. doi: 10.2135/cropsci1998.0011183X003800030028x

[ref70] Evans D. R. Hill J. Williams T. A. Rhodes I. (1985). Effects of coexistence on the performance of white clover-perennial ryegrass mixtures. Oecologia 66, 536–539. doi: 10.1007/BF00379346, PMID: 28310795

[ref71] Fan Y. Wang Z. Liao D. Raza M. A. Wang B. Zhang J.. (2020). Uptake and utilization of nitrogen, phosphorus and potassium as related to yield advantage in maize-soybean intercropping under different row configurations. Sci. Rep. 10:9504. doi: 10.1038/s41598-020-66459-y 32528144 PMC7290029

[ref72] Fargione J. Tilman D. (2005). Niche differences in phenology and rooting depth promote coexistence with a dominant C4 bunchgrass. Oecologia 143, 598–606. doi: 10.1007/s00442-005-0010-y, PMID: 15791430

[ref73] Favre J. R. Castiblanco T. M. Combs D. K. Wattiaux M. A. Picasso V. D. (2019). Forage nutritive value and predicted fiber digestibility of Kernza intermediate wheatgrass in monoculture and in mixture with red clover during the first production year. Anim. Feed Sci. Technol. 258:114298. doi: 10.1016/j.anifeedsci.2019.114298

[ref74] Finn J. A. Kirwan L. Connolly J. Sebastià M. T. Helgadottir A. Baadshaug O. H.. (2013). Ecosystem function enhanced by combining four functional types of plant species in intensively managed grassland mixtures: a 3-year continental-scale field experiment. J. Appl. Ecol. 50, 365–375. doi: 10.1111/1365-2664.12041

[ref75] Flynn E. S. Moore K. J. Singer J. W. Lamkey K. R. (2013). Evaluation of grass and legume species as perennial ground covers in corn production. Crop Sci. 53, 611–620. doi: 10.2135/cropsci2011.06.0306

[ref76] Foley J. A. DeFries R. Asner G. P. Barford C. Bonan G. Carpenter S. R.. (2005). Global consequences of land use. Science 309, 570–574. doi: 10.1126/science.1111772, PMID: 16040698

[ref77] Food and Agriculture Organization (FAO) (2019). FAOSTAT Statistical Database of the United Nation Food and Agriculture Organization (FAO) Statistical Division. Rome.

[ref78] Forcella F. Patel S. Lenssen A. W. Hoerning C. Wells M. S. Gesch R. W.. (2021). Weather and landscape influences on pollinator visitation of flowering winter oilseeds (field pennycress and winter camelina). J. Appl. Entomol. 145, 286–294. doi: 10.1111/jen.12854

[ref79] Frankow-Lindberg B. E. Brophy C. Collins R. P. Connolly J. (2009). Biodiversity effects on yield and unsown species invasion in a temperate forage ecosystem. Ann. Bot. 103, 913–921. doi: 10.1093/aob/mcp008, PMID: 19168861 PMC2707887

[ref80] Frankow-Lindberg B. E. Dahlin A. S. (2013). N2 fixation, N transfer, and yield in grassland communities including a deep-rooted legume or non-legume species. Plant Soil 370, 567–581. doi: 10.1007/s11104-013-1650-z, PMID: 35270112

[ref81] Frels K. Chopra R. Dorn K. M. Wyse D. L. Marks M. D. Anderson J. A. (2019). Genetic diversity of field pennycress (*Thlaspi arvense*) reveals untapped variability and paths toward selection for domestication. Agronomy 9:302. doi: 10.3390/agronomy9060302

[ref82] Gaba S. Lescourret F. Boudsocq S. Enjalbert J. Hinsinger P. Journet E.-P.. (2015). Multiple cropping systems as drivers for providing multiple ecosystem services: from concepts to design. Agron. Sustain. Dev. 35, 607–623. doi: 10.1007/s13593-014-0272-z

[ref83] Gasol C. M. Gabarrell X. Anton A. Rigola M. Carrasco J. Ciria P.. (2007). Life cycle assessment of a *Brassica carinata* bioenergy cropping system in southern Europe. Biomass Bioenergy 31, 543–555. doi: 10.1016/j.biombioe.2007.01.026

[ref84] Gaudio N. Escobar-Gutiérrez A. J. Casadebaig P. Evers J. B. Gérard F. Louarn G.. (2019). Current knowledge and future research opportunities for modeling annual crop mixtures. A review. Agron. Sustain. Dev. 39:20. doi: 10.1007/s13593-019-0562-6

[ref85] Georges M. Charlier C. Hayes B. (2019). Harnessing genomic information for livestock improvement. Nat. Rev. Genet. 20, 135–156. doi: 10.1038/s41576-018-0082-2, PMID: 30514919

[ref86] Gesch R. W. Archer D. W. Berti M. T. (2014). Dual cropping winter camelina with soybean in the northern corn belt. Agron. J. 106, 1735–1745. doi: 10.2134/agronj14.0215

[ref87] Gesch R. W. Johnson J. M. F. (2015). Water use in camelina–soybean dual cropping systems. Agron. J. 107, 1098–1104. doi: 10.2134/agronj14.0626, PMID: 34200509

[ref88] Ginakes P. Grossman J. Baker J. Sooksa-nguan T. (2020). Tillage intensity influences nitrogen cycling in organic kura clover living mulch. Nutr. Cycl. Agroecosyst. 116, 71–82. doi: 10.1007/s10705-019-10025-0

[ref89] Grabber J. H. Jokela W. E. (2013). Off-season groundcover and runoff characteristics of perennial clover and annual grass companion crops for no-till corn fertilized with manure. J. Soil Water Conserv. 68, 411–418. doi: 10.2489/jswc.68.5.411

[ref90] Grieder C. Kempf K. Schubiger F. X. (2021). Breeding alfalfa (*Medicago sativa* L.) in mixture with grasses. Sustain. For. 13:8929. doi: 10.3390/su13168929

[ref91] Griffing B. (1956). Concept of general and specific combining ability in relation to diallel crossing systems. Aust. J. Biol. Sci. 9, 463–493. doi: 10.1071/BI9560463

[ref92] Hamblin J. Rowell J. G. Redden R. (1976). Selection for mixed cropping. Euphytica 25, 97–106. doi: 10.1007/BF00041533, PMID: 34925421

[ref93] Haug B. Messmer M. M. Enjalbert J. Goldringer I. Forst E. Flutre T.. (2021). Advances in breeding for mixed cropping – incomplete factorials and the producer/associate concept. Front. Plant Sci. 11:620400. doi: 10.3389/fpls.2020.620400, PMID: 33505418 PMC7829252

[ref94] Hauggaard-Nielsen H. Ambus P. Jensen E. S. (2001). Interspecific competition, N use and interference with weeds in pea–barley intercropping. Field Crop Res. 70, 101–109. doi: 10.1016/S0378-4290(01)00126-5

[ref95] Hauggaard-Nielsen H. Jensen E. S. (2005). Facilitative root interactions in intercrops. Plant Soil 274, 237–250. doi: 10.1007/s11104-004-1305-1

[ref96] Haynes R. J. (1980). Competitive Aspects of the Grass-Legume Association. Adv. Agron. 33, 227–261. doi: 10.1016/S0065-2113(08)60168-6

[ref97] Hector A. Schmid B. Beierkuhnlein C. Caldeira M. C. Diemer M. Dimitrakopoulos P. G.. (1999). Plant diversity and productivity experiments in European grasslands. Science 286, 1123–1127. doi: 10.1126/science.286.5442.1123, PMID: 10550043

[ref98] Henkhaus N. Bartlett M. Gang D. Grumet R. Jordon-Thaden I. Lorence A.. (2020). Plant science decadal vision 2020–2030: reimagining the potential of plants for a healthy and sustainable future. Plant Direct 4, 1–24. doi: 10.1002/pld3.252 PMC7459197 32904806

[ref99] Hesketh J. D. Myhre D. L. Willey C. R. (1973). Temperature control of time intervals Between vegetative and reproductive events in Soybeans1. Crop Sci. 13, 250–254. doi: 10.2135/cropsci1973.0011183X001300020030x

[ref100] Hickey J. M. Chiurugwi T. Mackay I. Powell W. (2017). Genomic prediction unifies animal and plant breeding programs to form platforms for biological discovery. Nat. Genet. 49, 1297–1303. doi: 10.1038/ng.3920, PMID: 28854179

[ref101] Hill J. (1990). The three C’s - competition, coexistence and coevolution - and their impact on the breeding of forage crop mixtures. Theor. Appl. Genet. 79, 168–176. doi: 10.1007/BF00225947, PMID: 24226214

[ref102] Hill J. (1996). Breeding components for mixture performance. Euphytica 92, 135–138. doi: 10.1007/BF00022838, PMID: 34579296

[ref103] Hill J. Michaelson-Yeates T. P. T. (1987). Effects of competition upon the productivity of white clover-perennial ryegrass mixtures. Analysis of and interrelations between characters. Plant Breed. 98, 161–170. doi: 10.1111/j.1439-0523.1987.tb01110.x

[ref104] Hoerning C.A. (2019). Analysis of Crop-Competition, Weeds, and Heterodera Glycines in Winter Annual Oilseed Rotation. Doctoral dissertation. University of Minnesota.

[ref105] Hoerning C. Wells M. S. Gesch R. Forcella F. Wyse D. (2020). Yield tradeoffs and weed suppression in a winter annual oilseed relay-cropping system. Agron. J. 112, 2485–2495. doi: 10.1002/agj2.20160

[ref106] Holland J. B. Brummer E. C. (1999). Cultivar effects on oat–berseem clover intercrops. Agron. J. 91, 321–329. doi: 10.2134/agronj1999.00021962009100020023x

[ref107] Honig J. A. Averello V. Bonos S. A. Meyer W. A. (2012). Classification of Kentucky bluegrass (*Poa pratensis* L.) cultivars and accessions based on microsatellite (simple sequence repeat) markers. HortScience 47, 1356–1366. doi: 10.21273/HORTSCI.47.9.1356

[ref108] Hussain S. Pang T. Iqbal N. Shafiq I. Skalicky M. Brestic M.. (2020). Acclimation strategy and plasticity of different soybean genotypes in intercropping. Funct. Plant Biol. 47, 592–610. doi: 10.1071/FP19161, PMID: 32375994

[ref109] Hyder D. N. (1972). “Defoliation in relation to vegetative growth,” in The Biology and Utilization of Grasses. eds. Youngner V. B. McKell C. M. (New York: Academic Press), 302–317.

[ref110] Johnson W. G. Earl Creech J. Mock V. A. (2008). Role of winter annual weeds as alternative hosts for soybean cyst nematode. Crop Manag. 7, 1–9. doi: 10.1094/CM-2008-0701-01-RV

[ref111] Johnson G. A. Wells M. S. Anderson K. Gesch R. W. Forcella F. Wyse D. L. (2017). Yield tradeoffs and nitrogen between pennycress, Camelina, and soybean in relay- and double-crop systems. Agron. J. 109, 2128–2135. doi: 10.2134/agronj2017.02.0065

[ref112] Jones T. A. Carlson I. T. Buxton D. R. (1989). Legume compatibility of reed canarygrass clones related to agronomic and other morphological traits. Crop Sci. 29, 1–248. doi: 10.2135/cropsci1989.0011183X002900010001x

[ref113] Jungers J. M. DeHaan L. R. Betts K. J. Sheaffer C. C. Wyse D. L. (2017). Intermediate wheatgrass grain and forage yield responses to nitrogen fertilization. Agron. J. 109, 462–472. doi: 10.2134/agronj2016.07.0438

[ref114] Jungers J. M. Kaiser D. E. Lamb J. F. S. Lamb J. A. Noland R. L. Samac D. A.. (2019). Potassium fertilization affects alfalfa forage yield, nutritive value, root traits, and persistence. Agron. J. 111, 2843–2852. doi: 10.2134/agronj2019.01.0011

[ref115] Kauffmann K. D. Dudley J. W. (1979). Selection indices for corn grain yield, percent protein, and kernel weight 1. Crop Sci. 19, 583–588. doi: 10.2135/cropsci1979.0011183X001900050008x

[ref116] Keller W. (1946). Designs and technic for the adaptation of controlled competition to forage plant breeding1. Agron. J. 38, 580–588. doi: 10.2134/agronj1946.00021962003800070002x

[ref117] Knowles R. P. (1977). Recurrent mass selection for improved seed yields in intermediate Wheatgrass1. Crop Sci. 17, 51–54. doi: 10.2135/cropsci1977.0011183X001700010015x

[ref118] Lamp W. O. (1991). Reduced Empoasca fabae (Homoptera: Cicadellidae) density in oat–alfalfa intercrop systems. Environ. Entomol. 20, 118–126. doi: 10.1093/ee/20.1.118

[ref119] Lee D. Owens V. N. Boe A. Koo B.-C. (2009). Biomass and seed yields of big bluestem, switchgrass, and intermediate wheatgrass in response to manure and harvest timing at two topographic positions. GCB Bioenergy 1, 171–179. doi: 10.1111/j.1757-1707.2009.01008.x

[ref120] Letourneau D. K. Armbrecht I. Rivera B. S. Lerma J. M. Carmona E. J. Daza M. C.. (2011). Does plant diversity benefit agroecosystems? A synthetic review. Ecol. Appl. 21, 9–21. doi: 10.1890/09-2026.1, PMID: 21516884

[ref121] Li C. Hoffland E. Kuyper T. W. Yu Y. Zhang C. Li H.. (2020). Syndromes of production in intercropping impact yield gains. Nat. Plants 6, 653–660. doi: 10.1038/s41477-020-0680-9, PMID: 32483328

[ref122] Li L. Li S.-M. Sun J.-H. Zhou L.-L. Bao X.-G. Zhang H.-G.. (2007). Diversity enhances agricultural productivity via rhizosphere phosphorus facilitation on phosphorus-deficient soils. PNAS 104, 11192–11196. doi: 10.1073/pnas.0704591104, PMID: 17592130 PMC1899187

[ref123] Li X. Mu Y. Cheng Y. Liu X. Nian H. (2013). Effects of intercropping sugarcane and soybean on growth, rhizosphere soil microbes, nitrogen and phosphorus availability. Acta Physiol. Plant. 35, 1113–1119. doi: 10.1007/s11738-012-1148-y

[ref124] Li L. Sun J. Zhang F. Li X. Yang S. Rengel Z. (2001). Wheat/maize or wheat/soybean strip intercropping: I. yield advantage and interspecific interactions on nutrients. Field Crop Res. 71, 123–137. doi: 10.1016/S0378-4290(01)00156-3, PMID: 35167890

[ref125] Li L. Tilman D. Lambers H. Zhang F.-S. (2014). Plant diversity and overyielding: insights from belowground facilitation of intercropping in agriculture. New Phytol. 203, 63–69. doi: 10.1111/nph.12778, PMID: 25013876

[ref126] Li X. F. Wang Z. G. Bao X. G. Sun J.-H. Yang S.-C. Wang P.. (2021). Long-term increased grain yield and soil fertility from intercropping. Nat. Sustain. 9, 943–950. doi: 10.1038/s41893-021-00767-7

[ref127] Litrico I. Violle C. (2015). Diversity in plant breeding: a new conceptual framework. Trends Plant Sci. 20, 604–613. doi: 10.1016/j.tplants.2015.07.007, PMID: 26440430

[ref128] Lollato R. P. Marburger D. Holman J. D. Tomlinson P. Presley D. Edwards J. T. (2017). Dual-Purpose Wheat: Management for Forage and Grain Production (No. MF3375). Kansas State University Agricultural Experiment Station and Cooperative Extension Service.

[ref129] Loreau M. Hector A. (2001). Partitioning selection and complementarity in biodiversity experiments. Nature 412, 72–76. doi: 10.1038/35083573, PMID: 11452308

[ref130] Maamouri A. Louarn G. Béguier V. Julier B. (2017). Performance of lucerne genotypes for biomass production and nitrogen content differs in monoculture and in mixture with grasses and is partly predicted from traits recorded on isolated plants. Crop Pasture Sci. 68, 942–951. doi: 10.1071/CP17052

[ref131] Maamouri A. Louarn G. Gastal F. Béguier V. Julier B. Maamouri A.. (2015). Effects of lucerne genotype on morphology, biomass production and nitrogen content of lucerne and tall fescue in mixed pastures. Crop Pasture Sci. 66, 192–204. doi: 10.1071/CP14164

[ref132] Majak W. Garland G. J. Lysyk T. J. (2003). The effect of herbage mixtures of alfalfa and orchardgrass on the incidence of bloat in cattle. Can. J. Anim. Sci. 83, 827–829. doi: 10.4141/A03-078

[ref133] Malézieux E. Crozat Y. Dupraz C. Laurans M. Makowski D. Ozier-Lafontaine H.. (2009). Mixing plant species in cropping systems: concepts, tools and models. A review. Agron. Sustain. Dev. 29, 43–62. doi: 10.1051/agro:2007057

[ref134] Malhi S. S. Zentner R. P. Heier K. (2002). Effectiveness of alfalfa in reducing fertilizer N input for optimum forage yield, protein concentration, returns and energy performance of bromegrass-alfalfa mixtures. Nutr. Cycl. Agroecosyst. 62, 219–227. doi: 10.1023/A:1021229824357

[ref135] Marks M. D. Chopra R. Sedbrook J. C. (2021). Technologies enabling rapid crop improvements for sustainable agriculture: example pennycress (*Thlaspi arvense* L.). Emerg. Top. Life Sci. 5, 325–335. doi: 10.1042/ETLS20200330, PMID: 33755137

[ref136] Marquard E. Weigelt A. Temperton V. M. Roscher C. Schumacher J. Buchmann N.. (2009). Plant species richness and functional composition drive overyielding in a six-year grassland experiment. Ecology 90, 3290–3302. doi: 10.1890/09-0069.1, PMID: 20120799

[ref137] Martin M. P. L. D. Field R. J. (1984). The nature of competition between perennial ryegrass and white clover. Grass Forage Sci. 39, 247–253. doi: 10.1111/j.1365-2494.1984.tb01689.x

[ref138] Martin R. C. Greyson P. R. Gordon R. (1999). Competition between corn and a living mulch. Can. J. Plant Sci. 79, 579–586. doi: 10.4141/P98-089

[ref139] Martin-Guay M. O. Paquette A. Dupras J. Rivest D. (2018). The new green revolution: sustainable intensification of agriculture by intercropping. Sci. Total Environ. 615, 767–772. doi: 10.1016/j.scitotenv.2017.10.024, PMID: 28992501

[ref140] McCabe G. J. Wolock D. M. (2010). Long-term variability in northern hemisphere snow cover and associations with warmer winters. Clim. Chang. 99, 141–153. doi: 10.1007/s10584-009-9675-2

[ref141] McElroy M. S. Papadopoulos Y. A. Adl M. S. (2012). Complexity and composition of pasture swards affect plant productivity and soil organisms. Can. J. Plant Sci. 92, 687–697. doi: 10.4141/cjps2011-147

[ref142] Mead R. Willey R. W. (1980). The concept of a ‘land equivalent ratio’ and advantages in yields from intercropping. Exp. Agric. 16, 217–228. doi: 10.1017/S0014479700010978

[ref143] Menchaca L. Connolly J. (1990). Species interference in white clover-ryegrass mixtures. J. Ecol. 78, 223–232. doi: 10.2307/2261047

[ref144] Messina C. McDonald D. Poffenbarger H. Clark R. Salinas A. Fang Y.. (2021). Reproductive resilience but not root architecture underpins yield improvement under drought in maize. J. Exp. Bot. 72, 5235–5245. doi: 10.1093/jxb/erab231, PMID: 34037765 PMC8272564

[ref145] Meuwissen T. H. Hayes B. J. Goddard M. E. (2001). Prediction of total genetic value using genome-wide dense marker maps. Genetics 157, 1819–1829. doi: 10.1093/genetics/157.4.1819, PMID: 11290733 PMC1461589

[ref146] Moore K. J. Anex R. P. Elobeid A. E. Fei S. Flora C. B. Goggi A. S.. (2019). Regenerating agricultural landscapes with perennial groundcover for intensive crop production. Agronomy 9:458. doi: 10.3390/agronomy9080458

[ref147] Moore D. R. E. Waid J. S. (1971). The influence of washings of living roots on nitrification. Soil Biol. Biochem. 3, 69–83. doi: 10.1016/0038-0717(71)90032-0

[ref148] Mortensen D. A. Smith R. G. (2020). Confronting barriers to cropping system diversification. Front. Sustain. Food Syst. 4:199. doi: 10.3389/fsufs.2020.564197

[ref149] Mortenson J. Waldron B. Larson S. Jensen K. DeHaan L. Peel M.. (2019). Quantitative trait loci (QTL) for forage traits in intermediate wheatgrass when grown as spaced-plants versus monoculture and polyculture swards. Agronomy 9:580. doi: 10.3390/agronomy9100580

[ref150] Nyfeler D. Huguenin-Elie O. Suter M. Frossard E. Connolly J. Lüscher A. (2009). Strong mixture effects among four species in fertilized agricultural grassland led to persistent and consistent transgressive overyielding. J. Appl. Ecol. 46, 683–691. doi: 10.1111/j.1365-2664.2009.01653.x

[ref151] Nyfeler D. Huguenin-Elie O. Suter M. Frossard E. Luescher A. (2011). Grass-legume mixtures can yield more nitrogen than legume pure stands due to mutual stimulation of nitrogen uptake from symbiotic and non-symbiotic sources. Agric. Ecosyst. Environ. 140, 155–163. doi: 10.1016/j.agee.2010.11.022

[ref152] Ofir M. Kigel J. (1999). Photothermal control of the imposition of summer dormancy in *Poa bulbosa*, a perennial grass geophyte. Physiol. Plant. 105, 633–640. doi: 10.1034/j.1399-3054.1999.105406.x

[ref153] Ogle D. G. St. John L. Jensen K. B. (2011). Intermediate Wheatgrass. USDA NRCS. Available at: https://www.nrcs.usda.gov/Internet/FSE_PLANTMATERIALS/publications/idpmspg04843.pdf (Accessed April 9, 2021).

[ref154] Ott M. A. Eberle C. A. Thom M. D. Archer D. W. Forcella F. Gesch R. W.. (2019). Economics and agronomics of relay-cropping pennycress and camelina with soybean in Minnesota. Agron. J. 111, 1281–1292. doi: 10.2134/agronj2018.04.0277

[ref155] Papadopoulos Y. A. McElroy M. S. Fillmore S. A. E. McRae K. B. Duyinsveld J. L. Fredeen A. H. (2012). Sward complexity and grass species composition affect the performance of grass-white clover pasture mixtures. Can. J. Plant Sci. 92, 1199–1205. doi: 10.4141/cjps2012-015

[ref156] Peltier A. J. Bradley C. A. Chilvers M. I. Malvick D. K. Mueller D. S. Wise K. A.. (2012). Biology, yield loss and control of Sclerotinia stem rot of soybean. J. Integrat. Pest Manag. 3, B1–B7. doi: 10.1603/IPM11033

[ref157] Picasso V. D. Brummer E. C. Liebman M. Dixon P. M. Wilsey B. J. (2008). Crop species diversity affects productivity and weed suppression in perennial polycultures under two management strategies. Crop Sci. 48, 331–342. doi: 10.2135/cropsci2007.04.0225

[ref158] Picasso V. D. Brummer E. C. Liebman M. Dixon P. M. Wilsey B. J. (2011). Diverse perennial crop mixtures sustain higher productivity over time based on ecological complementarity. Renew. Agric. Food Syst. 26, 317–327. doi: 10.1017/S1742170511000135

[ref159] Pretty J. Benton T. G. Bharucha Z. P. Dicks L. V. Flora C. B. Godfray H. C. J.. (2018). Global assessment of agricultural system redesign for sustainable intensification. Nat. Sustain. 1, 441–446. doi: 10.1038/s41893-018-0114-0

[ref160] Pugliese J. Y. Culman S. W. Sprunger C. D. (2019). Harvesting forage of the perennial grain crop kernza (*Thinopyrum intermedium*) increases root biomass and soil nitrogen cycling. Plant Soil 437, 241–254. doi: 10.1007/s11104-019-03974-6

[ref161] Putnam A. R. DeFrank J. Barnes J. P. (1983). Exploitation of allelopathy for weed control in annual and perennial cropping systems. J. Chem. Ecol. 9, 1001–1010. doi: 10.1007/BF00982207, PMID: 24407796

[ref162] Rajcan I. Chandler K. J. Swanton C. J. (2004). Red–far-red ratio of reflected light: a hypothesis of why early-season weed control is important in corn. Weed Sci. 52, 774–778. doi: 10.1614/WS-03-158R

[ref163] Rao M. R. Willey R. W. (1980). Evaluation of yield stability in intercropping: studies on Sorghum/Pigeonpea. Exp. Agric. 16, 105–116. doi: 10.1017/S0014479700010796

[ref164] Raseduzzaman M. Jensen E. S. (2017). Does intercropping enhance yield stability in arable crop production? A meta-analysis. Eur. J. Agron. 91, 25–33. doi: 10.1016/j.eja.2017.09.009

[ref165] Raskin D. Wells M. S. Grossman J. M. Coulter J. A. Sheaffer C. C. (2017). Yield and economic potential of spring-planted, pea–barley forage in short-season corn double-crop systems. Agron. J. 109, 2486–2498. doi: 10.2134/agronj2017.01.0029

[ref166] Rasmussen J. Søegaard K. Pirhofer-Walzl K. Eriksen J. (2012). N2-fixation and residual N effect of four legume species and four companion grass species. Eur. J. Agron. 36, 66–74. doi: 10.1016/j.eja.2011.09.003

[ref167] Reiss E. R. Drinkwater L. E. (2018). Cultivar mixtures: a meta-analysis of the effect of intraspecific diversity on crop yield. Ecol. Appl. 28, 62–77. doi: 10.1002/eap.1629, PMID: 28940830

[ref168] Riday H. Brummer E. C. (2014). Vigor and persistence of Birdsfoot trefoil populations selected with or without an Orchardgrass companion evaluated in grass sod. Crop Sci. 54, 2070–2076. doi: 10.2135/cropsci2014.02.0147

[ref169] Roda A. L. Landis D. A. Coggins M. L. Spandl E. Hesterman O. B. (1996). Forage grasses decrease alfalfa weevil (Coleoptera: Curculionidae) damage and larval numbers in alfalfa-grass intercrops. J. Econ. Entomol. 89, 743–750. doi: 10.1093/jee/89.3.743

[ref170] Rowe D. E. Brink G. E. (1993). Heritabilities and genetic correlations of white clover clones grown in three environments. Crop Sci. 33, 1149–1152. doi: 10.2135/cropsci1993.0011183X003300060008x

[ref171] Rumbaugh M. D. Pendery B. M. (1991). Stability of forage yield of alfalfa clones grown with five associate species. Can. J. Plant Sci. 71, 453–459. doi: 10.4141/cjps91-062

[ref172] Ryan M. R. Crews T. E. Culman S. W. DeHaan L. R. Hayes R. C. Jungers J. M.. (2018). Managing for multifunctionality in perennial grain crops. Bioscience 68, 294–304. doi: 10.1093/biosci/biy014, PMID: 29662249 PMC5894082

[ref173] Sampoux J.-P. Giraud H. Litrico I. (2020). Which recurrent selection scheme to improve mixtures of crop species? Theoretical expectations. G3 10, 89–107. doi: 10.1534/g3.119.400809 31672848 PMC6945008

[ref174] Sánchez B. Rasmussen A. Porter J. R. (2014). Temperatures and the growth and development of maize and rice: a review. Glob. Chang. Biol. 20, 408–417. doi: 10.1111/gcb.12389, PMID: 24038930

[ref175] Sanders Z. P. Andrews J. S. Saha U. K. Vencill W. Lee R. D. Hill N. S. (2017). Optimizing agronomic practices for clover persistence and corn yield in a white clover-corn living mulch system. Agron. J. 109, 2025–2032. doi: 10.2134/agronj2017.02.0106

[ref176] Sanderson M. A. Brink G. Ruth L. Stout R. (2012). Grass–legume mixtures suppress weeds during establishment better than monocultures. Agron. J. 104, 36–42. doi: 10.2134/agronj2011.0130

[ref177] Sanderson M. A. Brink G. Stout R. Ruth L. (2013). Grass–legume proportions in forage seed mixtures and effects on herbage yield and weed abundance. Agron. J. 105, 1289–1297. doi: 10.2134/agronj2013.0131

[ref178] Saudy H. El-Metwally I. (2009). Weed management under different patterns of sunflower-soybean intercropping. J. Cent. Eur. Agric. 10, 41–51.

[ref179] Schlautman B. Bartel C. Diaz-Garcia L. Fei S. Flynn S. Haramoto E.. (2021). Perennial groundcovers: an emerging technology for soil conservation and the sustainable intensification of agriculture. Emerg. Top. Life Sci. 5, 337–347. doi: 10.1042/ETLS20200318, PMID: 33973632 PMC8166338

[ref180] Schoener T. W. (1983). Field experiments on interspecific competition. Am. Nat. 122, 240–285. doi: 10.1086/284133, PMID: 35276736

[ref181] Séguin-Swartz G. Eynck C. Gugel R. K. Strelkov S. E. Olivier C. Y. Li J. L.. (2009). Diseases of *Camelina sativa* (false flax). Can. J. Plant Pathol. 31, 375–386. doi: 10.1080/07060660909507612, PMID: 34844449

[ref182] Short K. E. Carlson I. T. (1989). Bidirectional selection for birdsfoot trefoil-compatibility traits in Orchardgrass. Crop Sci. 29, 1131–1136. doi: 10.2135/cropsci1989.0011183X002900050006x

[ref183] Sleugh B. Moore K. J. George J. R. Brummer E. C. (2000). Binary legume-grass mixtures improve forage yield, quality, and seasonal distribution. Agron. J. 92, 24–29. doi: 10.2134/agronj2000.92124x

[ref184] Smith T. J. Camper H. M. Jr. (1975). Effects of seed size on soybean performance. Agron. J. 67, 681–684. doi: 10.2134/agronj1975.00021962006700050025x, PMID: 35275252

[ref185] Song X. M. Wang J. P. Sun P. C. Ma X. Yang Q. H. Hu J. J.. (2020). Preferential gene retention increases the robustness of cold regulation in Brassicaceae and other plants after polyploidization. Hortic. Res. 7:20. doi: 10.1038/s41438-020-0253-0, PMID: 32133148 PMC7035258

[ref186] Sprague G. F. Tatum L. A. (1942). General vs. specific combining ability in single crosses of Corn1. Agron. J. 34, 923–932. doi: 10.2134/agronj1942.00021962003400100008x

[ref187] Sturludóttir E. Brophy C. Bélanger G. Gustavsson A.-M. Jørgensen M. Lunnan T.. (2014). Benefits of mixing grasses and legumes for herbage yield and nutritive value in Northern Europe and Canada. Grass Forage Sci. 69, 229–240. doi: 10.1111/gfs.12037

[ref188] Subbarao G. V. Kishii M. Bozal-Leorri A. Ortiz-Monasterio I. Gao X. Ibba M. I.. (2021). Enlisting wild grass genes to combat nitrification in wheat farming: a nature-based solution. Proc. Natl. Acad. Sci. 118:e2106595118. doi: 10.1073/pnas.2106595118, PMID: 34426500 PMC8536370

[ref189] Subbarao G. V. Nakahara K. Hurtado M. P. Ono H. Moreta D. E. Salcedo A. F.. (2009). Evidence for biological nitrification inhibition in Brachiaria pastures. Proc. Natl. Acad. Sci. U. S. A. 41, 17302–17307. doi: 10.1073/pnas.0903694106, PMID: 19805171 PMC2752401

[ref190] Subbarao G. V. Rondon M. Ito O. Ishikawa T. Rao I. M. Nakahara K.. (2007). Biological nitrification inhibition (BNI)—is it a widespread phenomenon? Plant Soil 294, 5–18. doi: 10.1007/s11104-006-9159-3

[ref191] Suplick-Ploense M. R. Qian Y. (2005). Evapotranspiration, rooting characteristics, and dehydration avoidance: comparisons between hybrid bluegrass and Kentucky bluegrass. Int. Turfgrass Soc. Res. J. 10, 891–898.

[ref192] Tamburini G. Bommarco R. Wanger T. C. Kremen C. van der Heijden M. G. A. Liebman M.. (2020). Agricultural diversification promotes multiple ecosystem services without compromising yield. Sci. Adv. 6:eaba1715. doi: 10.1126/sciadv.aba1715, PMID: 33148637 PMC7673676

[ref193] Tautges N. E. Jungers J. M. DeHaan L. R. Wyse D. L. Sheaffer C. C. (2018). Maintaining grain yields of the perennial cereal intermediate wheatgrass in monoculture v. bi-culture with alfalfa in the Upper Midwestern USA. J. Agric. Sci. 156, 758–773. doi: 10.1017/S0021859618000680

[ref194] Temperton V. M. Mwangi P. N. Scherer-Lorenzen M. Schmid B. Buchmann N. (2007). Positive interactions between nitrogen-fixing legumes and four different neighbouring species in a biodiversity experiment. Oecologia 151, 190–205. doi: 10.1007/s00442-006-0576-z, PMID: 17048010

[ref195] Thomas R. J. (1992). The role of the legume in the nitrogen cycle of productive and sustainable pastures. Grass Forage Sci. 47, 133–142. doi: 10.1111/j.1365-2494.1992.tb02256.x

[ref196] Tracy B. F. Albrecht K. Flores J. Hall M. Islam A. Jones G.. (2016). Evaluation of Alfalfa–Tall fescue mixtures across multiple environments. Crop Sci. 56, 2026–2034. doi: 10.2135/cropsci2015.09.0553

[ref197] Trenbath B. R. (1974). “Biomass productivity of mixtures,” in Advances in Agronomy. ed. Brady N. C. (Academic Press), 177–210.

[ref198] Tsay J. S. Fukai S. Wilson G. L. (1988). Intercropping cassava with soybean cultivars of varying maturities. Field Crop Res. 19, 211–225. doi: 10.1016/0378-4290(88)90044-5

[ref199] Turkington R. (1989). The growth, distribution and neighbour relationships of *Trifolium Repens* in a permanent pasture. V. The coevolution of competitors. J. Ecol. 77, 717–733. doi: 10.2307/2260981

[ref200] Undersander D. Cosgove D. Cullen E. Grau C. Rice M. E. Renz M.. (2011). Alfalfa Management Guide. ed. L. Al-Amoodi (Madison, WI: American Society of Agronomy, Inc., Crop Science Society of America, Inc. Soil Science Society of America, Inc.).

[ref201] VanRaden P. M. (2020). Symposium review: how to implement genomic selection. J. Dairy Sci. 103, 5291–5301. doi: 10.3168/jds.2019-17684, PMID: 32331884

[ref202] Veira D. M. Lysyk T. J. Thompson D. J. Garland G. J. Majak W. (2010). Effect of grazing mixtures of alfalfa and orchardgrass grown in strips on the incidence of bloat in cattle. Can. J. Anim. Sci. 90, 109–112. doi: 10.4141/CJAS09077

[ref203] Verret V. Gardarin A. Pelzer E. Médiène S. Makowski D. Valantin-Morison M. (2017). Can legume companion plants control weeds without decreasing crop yield? A meta-analysis. Field Crop Res. 204, 158–168. doi: 10.1016/j.fcr.2017.01.010

[ref204] Wagoner P. A. (1989). The study of intermediate wheatgrass as a perennial grain crop: 1988 research summary. [Emmaus, PA]: Rodale Research Center, Rodale Press. Available at: https://hdl.handle.net/2027/coo.31924073236097?urlappend=%3Bsignon=swle:https://shibidp.cit.cornell.edu/idp/shibboleth (Accessed April 14, 2021).

[ref205] Wagoner P. (1990). Perennial grain. New use for intermediate wheatgrass. J. Soil Water Conserv. 45, 81–82.

[ref206] Waldron B. L. Peel M. D. Larson S. R. Mott I. W. Creech J. E. (2017). Tall fescue forage mass in a grass-legume mixture: predicted efficiency of indirect selection. Euphytica 213:67. doi: 10.1007/s10681-017-1856-x

[ref207] Wallace S. U. Whitwell T. Palmer J. H. Hood C. E. Hull S. A. (1992). Growth of relay intercropped soybean. Agron. J. 84, 968–973. doi: 10.2134/agronj1992.00021962008400060012x, PMID: 33779899

[ref208] Warwick S. I. (2011). “Brassicaceae in agriculture,” in Genetics and Genomics of the Brassicaceae. eds. R. Schmidt and I. Bancroft (Gatersleben, Germany/Colney, Norwich, UK: Springer), 33–65.

[ref209] Weyers S. L. Gesch R. W. Forcella F. Eberle C. A. Thom M. D. Matthees H. L.. (2021). Surface runoff and nutrient dynamics in cover crop-soybean systems in the Upper Midwest. J. Environ. Qual. 50, 158–171. doi: 10.1002/jeq2.20135, PMID: 33345349

[ref210] Weyers S. Thom M. Forcella F. Eberle C. Matthees H. Gesch R.. (2019). Reduced potential for nitrogen loss in cover crop–soybean relay systems in a cold climate. J. Environ. Qual. 48, 660–669. doi: 10.2134/jeq2018.09.0350, PMID: 31180428

[ref211] Wiggans D. R. Singer J. W. Moore K. J. Lamkey K. R. (2012a). Response of continuous maize with stover removal to living mulches. Agron. J. 104, 917–925. doi: 10.2134/agronj2011.0395

[ref212] Wiggans D. R. Singer J. W. Moore K. J. Lamkey K. R. (2012b). Maize water use in living mulch systems with Stover removal. Crop Sci. 52, 327–338. doi: 10.2135/cropsci2011.06.0316

[ref213] Wolfe M. D. Jannink J. L. Kantar M. B. Santantonio N. (2021). Multi-species genomics-enabled selection for improving agroecosystems across space and time. Front. Plant Sci. 12:665349. doi: 10.3389/fpls.2021.665349, PMID: 34249037 PMC8261054

[ref214] Worthington M. Reberg-Horton C. (2013). Breeding cereal crops for enhanced weed suppression: optimizing allelopathy and competitive ability. J. Chem. Ecol. 39, 213–231. doi: 10.1007/s10886-013-0247-6, PMID: 23385368

[ref215] Wright A. J. (1985). Selection for Improved Yield in Inter-Specific Mixtures or Intercrops. Theor. Appl. Genet. 69, 399–407. doi: 10.1007/BF00570909, PMID: 24253909

[ref216] Xie W. Robins J. G. Escribano S. Bushman B. S. Zhang X. (2014). Cultivar × binary mixture interaction effect on agronomic traits in orchardgrass. Grassl. Sci. 60, 104–111. doi: 10.1111/grs.12047

[ref217] Zannone L. Rotili P. Paoletti R. Scotti C. (1986). Experimental studies of grass-legume associations. Agronomie 6, 931–940. doi: 10.1051/agro:19861009

[ref218] Zarrough K. M. Nelson C. J. Coutts J. H. (1983). Relationship between tillering and forage yield of tall fescue. I. Yield1. Crop Sci. 23, 333–337. doi: 10.2135/cropsci1983.0011183X002300020036x

[ref219] Zemenchik R. A. Albrecht K. A. Schultz M. K. (2001). Nitrogen replacement values of Kura clover and birdsfoot trefoil in mixtures with cool-season grasses. Agron. J. 93, 451–458. doi: 10.2134/agronj2001.932451x

[ref220] Zhang C. J. Auer C. (2019). Overwintering assessment of camelina (*Camelina sativa*) cultivars and congeneric species in the northeastern US. Ind. Crop. Prod. 139:111532. doi: 10.1016/j.indcrop.2019.111532

[ref221] Zhu J. van der Werf W. Anten N. P. R. Vos J. Evers J. B. (2015). The contribution of phenotypic plasticity to complementary light capture in plant mixtures. New Phytol. 207, 1213–1222. doi: 10.1111/nph.13416, PMID: 25898768

[ref222] Ziyomo C. Albrecht K. Baker J. Bernardo R. (2013). Corn performance under managed drought stress and in a Kura clover living mulch intercropping system. Agron. J. 105:579. doi: 10.2134/agronj2012.0427

